# A New Frontier in Food Safety: Cold Plasma Strategies for Effective Control of Fungi and Mycotoxins

**DOI:** 10.3390/toxins18060241

**Published:** 2026-05-23

**Authors:** Eva María Mateo, Fernando Mateo, Andrea Tarazona, María Ángeles García-Esparza, José Miguel Soria, Misericordia Jiménez

**Affiliations:** 1 Department of Microbiology and Ecology, Faculty of Medicine and Dentistry, University of Valencia, 46010 Valencia, Spain; 2 Department of Electronic Engineering, ETSE, University of Valencia, Burjassot, 46100 Valencia, Spain; fernando.mateo@uv.es; 3 Department of Microbiology and Ecology, Faculty of Biology, University of Valencia, Burjassot, 46100 Valencia, Spain; andrea.tarazona@uv.es; 4 Department of Pharmacy, Cardenal Herrera University-CEU Universities, 46001 Valencia, Spain; maria.garcia2@uchceu.es; 5 Department of Biomedical Sciences, Cardenal Herrera University-CEU Universities, 46001 Valencia, Spain; jose.soria@uchceu.es

**Keywords:** cold plasma, toxigenic fungi, mycotoxins, detoxification, nuts, dried fruits, cereal grains

## Abstract

Mycotoxins are compounds produced by the secondary metabolism of certain fungi. These compounds contaminate foods worldwide and pose a severe threat to the health of humans and animals. They also cause huge economic losses. A plethora of methodologies, encompassing agricultural, biological, chemical, and physical approaches, have been devised to curtail the presence of mycotoxins in food commodities. Among the physical processes, cold plasma (CP) has emerged as a useful technique for controlling the presence of toxigenic fungi in foods and for degrading the mycotoxins occurring in them without significantly affecting the quality and organoleptic properties of the treated commodities. The present review endeavors to demonstrate the efficacy of CP as a method of eradicating or reducing both the toxigenic mycobiota and the mycotoxins present in the most contaminated foods, including nuts, dried fruits, and cereal grains. The mechanisms of toxin degradation proposed by the different researchers are also examined and compared. Furthermore, the impact of the CP effect on the quality, sensorial characteristics, and toxicological properties of the treated food is thoroughly examined.

## 1. Introduction

Cold plasma (CP) has arisen as a promising non-thermal technology for improving food safety, particularly in low-moisture commodities susceptible to fungal colonization and mycotoxin contamination during storage and distribution [1,2,3,4]. CP generates reactive oxygen and nitrogen species, charged particles, and UV photons capable of inactivating microorganisms and degrading toxic metabolites at relatively low temperatures, thereby minimizing detrimental effects on food quality. Experimental studies have demonstrated the efficacy of CP against several toxigenic fungi, including *Aspergillus*, *Fusarium*, and *Alternaria* species, as well as its capacity to reduce mycotoxin levels in cereal grains, nuts, and dried foods [1,2,3,4,5,6,7,8,9,10]. However, treatment efficacy depends on the fungal species, the chemical structure of the target mycotoxin, the food matrix, and plasma operating conditions. Thus, a concise overview of the main toxigenic fungi and mycotoxins relevant to food safety is needed to contextualize the discussion of CP-based mitigation strategies.

Microscopic filamentous fungi cause food and feed spoilage. Some moldy foods pose a significant hazard to consumer health because of the fungal toxic metabolites, known as mycotoxins, produced during spoilage [11]. Toxigenic fungi can colonize plants in the field or they may grow on food products postharvest, remaining throughout the production chain under favorable environmental conditions [11]. These fungi may produce mycotoxins through secondary metabolic processes. These mycotoxins may persist along the food chain in final products even after the elimination of the producing fungi. If the mycotoxins are not reduced to safe levels, they may be present in the human or animal diet, posing a threat to their health by means of chronic or acute diseases. Mycotoxins can reach consumers directly through the intake of contaminated commodities or indirectly through the consumption of products of animal origin (milk, eggs, or meat) [12]. In most countries, the maximum concentrations of the most dangerous mycotoxins are regulated. The maximum allowable levels vary depending on the mycotoxin and the type of food. According to the Food and Agriculture Organization, 25% of the global food and feed production is contaminated by mycotoxins each year. This results in estimated losses of around 1 billion metric tons of food and food products [11].

Aflatoxins (AFs) are highly toxic and carcinogenic byproducts of aflatoxigenic *Aspergillus* spp. They contaminate cereal grains, nuts, and spices [13]. AFs can cause crop products to be unsuitable for consumption and pose a health risk to humans and livestock [14]. Aflatoxin B_1_ (AFB_1_) is the most prevalent naturally occurring toxicant in food crops and is mainly produced by strains of *Aspergillus parasiticus* and *A. flavus.* The International Agency for Research on Cancer (IARC) has classified AFB_1_ as a human carcinogen (Group 1) [15].

Other mycotoxins include the trichothecenes, particularly those classified as type-A and type-B trichothecenes. The most important examples of the former include T-2 toxin and HT-2 toxin, which are produced by various *Fusarium* species. The main type B-trichothecenes are deoxynivalenol (DON) and nivalenol (NIV), which are also produced by *Fusarium* species. They inhibit the synthesis of proteins, DNA, and RNA and are common contaminants of cereal grains. Type B-trichothecenes induce pathological lesions, including necrosis of the intestinal epithelium. Masked mycotoxins, such as DON-3-β-d-glucoside, are an emerging problem [16,17].

Zearalenone (ZEA) is a potent estrogenic metabolite produced mainly by *Fusarium* spp. that is involved in many mycotoxicoses in livestock, causing infertility and abortion. ZEA is a common contaminant of maize and other cereals [11,18] and is often associated with ZEA-14-glucoside, a “masked” mycotoxin [19].

Ochratoxin A (OTA) is the most toxic member of the ochratoxins. OTA exhibits multiple toxic effects including nephrotoxicity and carcinogenicity [15,20]. OTA-producing fungi belong to the genus *Aspergillus* and some species to the genus *Penicillium.* OTA has been detected in cereal grains, coffee beans, grapes, dried fruits, wine, beer, and cheese [13,21,22,23].

Fumonisin B_1_ (FB_1_) is the most relevant member of fumonisin type B produced by some *Fusarium* spp. and *Aspergillus* spp. [24,25]. FBs may cause leukoencephalomalacia in equines and also have toxic and carcinogenic effects. The IARC has classified them as probably carcinogenic (Group 2B) [15,18]. Fumonisins are found in maize and its derivatives, in other cereal grains, and in other food commodities [13,25,26].

Patulin (PAT) is a mycotoxin produced mainly by *Penicillium expansum* and *Aspergillus* spp., being frequently found in unfermented apple juice [18].

Alternariol and alternariol methyl ethers are mycotoxins produced by *Alternaria* spp. Fusaproliferin, moniliformin, beauvericin, and enniatins are emerging mycotoxins biosynthesized by some *Fusarium* spp. that can contaminate maize and adversely influence grain quality [27]. Ergot alkaloids are produced by certain members of the Clavicipitaceae and *Aspergillus fumigatus* and can impact livestock welfare through the consumption of contaminated tall fescue and may cause “ergotism” [28].

Various pre- and post-harvest strategies have been implemented to reduce fungal growth and mycotoxin contamination in foods. Some strategies for farming products begin in the field before harvest with the application of good agricultural practices (GAPs) that include approaches for the pre-harvest, harvest, and post-harvest phases. Pre-harvest actions include plowing, tilling, the selection of resistant varieties, and crop rotation [1,29,30,31,32]. Another methodology is hazard analysis and critical control point (HACCP), which applies mainly to post-harvest and processed food [33]. At harvest, it is crucial to choose the appropriate time and conditions that can help minimize fungal contamination.

During the post-harvest stage, GAPs focus heavily on controlling temperature and humidity and ensuring traceability to minimize contamination risks and preserve quality. GAPs are the primary line of defense against mycotoxin contamination, followed by the application of good manufacturing practices (GMPs) during the handling, storage, processing, and distribution of cereals [34]. The implementation of HACCP at this stage, including drying, sorting, etc. to remove impurities, is crucial to avoid mycotoxin contamination [35,36]. Possibly, in this phase, toxigenic fungi and mycotoxins may persist in food products. During postharvest and food processing, the approaches used for minimizing the load toxigenic fungi and mycotoxin content may be classified as: (a) chemical (use of ammonia, hydrogen peroxide, ozone, fungicides, etc.); (b) physical (dehulling, sorting, UV or gamma irradiation, high-pressure processing (HPP), pulsed electric field (PEF) processing, and thermal treatments); and (c) biological control (use of microbial antagonists, lactic acid bacteria, etc.) [1,37,38,39,40,41,42,43,44,45]. Despite the progress achieved by using these methods, there are several drawbacks to consider such as low efficacy, the need for expensive chemicals, and sophisticated equipment. Some methods are unfeasible, expensive, inefficient, and time-consuming [40,46]. HPP, PEF, electron beam irradiation, ultrasound, supercritical carbon dioxide, and cold plasma (CP) are new strategies that have demonstrated good potential for mycotoxin mitigation. Nevertheless, some physical methods have drawbacks such as long treatment time, low detoxification ability, and potential environmental pollution. Moreover, they may produce undesirable changes in the physicochemical and sensory properties of the treated foods, thus impacting their quality [47,48].

With increasing consumer demand for high-quality, fresh appearance, and long shelf life products, CP technologies are now being explored [46] as they hold strong antimicrobial activity, can degrade many mycotoxins, are environmentally friendly, efficient, easy to operate, and have a low impact on food appearance and nutritional quality [41,46,47,49].

This review focuses on CP as an emerging non-thermal technology for mitigating toxigenic fungi and mycotoxins in low-moisture foods. Particular attention is given to nuts, dried fruits, and cereal grains, which are among the commodities most susceptible to fungal contamination during storage and commercialization. This review comparatively analyzes the efficacy of CP for fungal inactivation and mycotoxin degradation across these food matrices, underscoring matrix-dependent responses and treatment limitations. Proposed degradation mechanisms involving reactive plasma species are critically discussed together with the effects of CP treatments on food quality, sensory characteristics, and toxicological safety. By integrating microbiological, chemical, and technological perspectives, this review identifies current knowledge gaps and future research needs for the industrial implementation of CP-based detoxification strategies. Although some reviews have discussed CP applications in food processing, this review specifically examines its use for the control of toxigenic fungi and mycotoxins in low-moisture food matrices, with an emphasis on degradation mechanisms and quality-related aspects.

## 2. The Nature of Cold Plasma, Devices, and Configurations

Cold plasma (CP) is a partially ionized gas composed of electrons, ions, atoms, and molecules in ground or excited states as well as reactive species such as radicals and metastable species. The overall system is quasi-neutral with a roughly zero net electrical charge. CP contains a wide range of energetic species and also emits ultraviolet (UV) photons and electromagnetic radiation. In nature, plasmas exist in the form of stars, which are examples of thermal or hot plasma due to their high temperature, or the aurora, an example of low-temperature plasma. Thermal plasma is in a state of thermodynamic equilibrium where all the species have the same high temperatures, while non-thermal plasma is in a state of non-equilibrium. The electrons in the non-thermal plasma have a temperature much higher than that of the heavy particles (ions, atoms, and molecules) [50].

In general, a plasma is generated by supplying sufficient energy to a gas to cause its ionization. CP is also mentioned in the literature concerning medical or food applications as low-temperature plasma (LTP) or non-thermal plasma (NTP); however, this is misleading. CP is a type of plasma that operates on the target at low temperature (the effluent gas is near ambient temperature), although the temperature of electrons in the gas where the plasma is generated is very high. It is partially ionized, meaning that only a small fraction of all atoms and molecules in the gas are ionized [51]. The bulk gas temperature of cold plasma remains close to ambient temperature under conditions used for food applications. It is abbreviated as CAP or LPCP, and it is not in a thermodynamic equilibrium state.

Although CP is a non-equilibrium system, it remains quasi-neutral, meaning that the densities of electrons and ions are approximately equal [50]. The maintenance of plasma requires a balance between the generation of charged particles through ionization and their loss through various mechanisms. Ionization may occur either directly from the ground state or via stepwise processes involving excited states. Charged particles are lost through recombination in the plasma volume as well as through diffusion to surrounding surfaces. The steady-state plasma density is therefore determined by the balance between these production and loss processes. The application of an electric field (or heat) to the gas disrupts that equilibrium, causing the existing ionized species (electrons and ions) to move, creating a low electrical current. If the electric field is weak, the low electrical current is negligible. If the intensity of the electric field increases by applying a high voltage, the speed of the electrons and ions following the electric field also increases, and new ions and electrons are generated. Applying a sufficient voltage causes the current to increase, leading to a breakdown of the gas and the formation of a significant number of active species, the majority of which are unstable. Atoms and molecules that have been exposed to intense energy may release excess energy in the form of electromagnetic radiation, including ultraviolet (UV) radiation, as they return to a more stable state. Plasma formation is the result of a variety of physical processes, each of which depends on the type of gas involved.

A thorough exposition of the theoretical foundations of plasma chemistry is found in Fridman’s work [52]. The presence of reactive species in plasma can be determined through the implementation of diverse experimental and instrumental methodologies that encompass techniques such as optical emission spectroscopy, mass spectroscopy, or optical absorption spectroscopy. In the case of the liquid phases, identification methods include electron paramagnetic resonance spectroscopy, UV–Vis absorption spectroscopy, or mass spectrometry [53].

When O_2_-based plasmas containing water vapor (with variable and system-dependent concentrations) are used as the plasma gas source, the active discharge region contains electrons, ions, excited species, and UV radiation, leading to the formation of a wide range of reactive oxygen species (ROS). These include oxygen radicals (•O), excited atomic oxygen (O^1^D), ground-state atomic oxygen (O(^3^p_j_)), hydroxyl radicals (•OH), hydroperoxyl radicals (•O_2_H), singlet oxygen molecules (^1^O_2_), superoxide radicals (•O_2_^−^), dioxygenyl cations (O_2_^+^), atomic oxygen cations (O^+^), atomic oxygen anions (O^−^), ozone (O_3_), and hydrogen peroxide (H_2_O_2_) [39,42,53,54,55]. Among these ROS, both short- and long-lived species are present. Short-lived species, such as •OH and O, typically persist for nanoseconds to microseconds and are mainly confined to the plasma discharge region or its immediate vicinity, while longer-lived species, including O_3_ and H_2_O_2_, are more stable and can diffuse beyond the plasma core and contribute to downstream chemical reactivity. These ROS exhibit strong oxidative properties and are primarily responsible for the effective inactivation of microorganisms.

When the CP is produced using air with a water vapor admixture, the plasma is constituted basically by ROS and reactive nitrogen species (RNS) in addition to UV radiation and charged particles. RNS formed in the discharge include atomic nitrogen (•N), metastable molecular nitrogen (•N_2_), molecular nitrogen ions (N_2_^+^, N_2_^−^), atomic nitrogen cations (N^+^), excited and metastable nitrogen molecules (N_2_*, N_2_(A^3^Σ_u_^+^)), and nitrogen oxides such as nitric oxide (NO) and nitrogen dioxide (NO_2_). As with ROS, some of these species are short-lived and exist predominantly within or near the plasma region. However, more stable molecular species (NO, N_2_O, NO_2_, N_2_O_3_, and NO_3_) are longer-lived species that can persist in the post-discharge region and contribute to antimicrobial and biochemical effects. The combined group of reactive oxygen and nitrogen species is commonly referred to as RONS [56].

When inert gases such as helium (He), argon (Ar), or neon (Ne) are used to generate plasma, the active discharge region is primarily composed of electrons, ions, and excited or metastable noble gas species. Due to the low chemical reactivity of these gases, the direct formation of RONS within the plasma core is limited. However, as the plasma effluent interacts with ambient air or moisture in the surrounding environment, energy transfer processes involving metastable species promote the formation of RONS in the post-discharge region or at the gas–liquid and gas–solid interfaces. Under these conditions, many short-lived species, such as hydroxyl radicals and atomic oxygen, are generated predominantly outside the plasma core, although their relative abundance depends on factors such as the gas composition, humidity, and discharge parameters [53,56].

In the presence of liquid water near the CP, these reactive species can produce plasma-activated water (PAW). The incorporation of RONS into the liquid phase imparts unique physicochemical properties, including decreased pH and increased electrical conductivity. Examples of RONS found in PAW include hydroxyl radicals, superoxide radicals, nitric oxide radicals (•NO), H_2_O_2_, O_3_, nitrate, nitrite, peroxynitrous acid (ONOOH), and peroxynitrite (ONOO^−^) [57,58]. These species are responsible for the antimicrobial activity and chemical reactivity of PAW.

The particular composition and relative abundance of RONS generated by CP depend on the plasma operating conditions, such as working gas composition, type of discharge, applied power, presence of water vapor or liquids, geometry, and post-discharge transport/exposure conditions [59].

The CP generation can be accomplished through a variety of techniques, including corona discharge, glow discharge, dielectric barrier discharge, plasma jet discharge, radio, and others [60,61]. Historically, the CP generation was constrained to low-pressure conditions, requiring the use of a vacuum chamber and thus limiting its applications. In recent years, plasma has been formed at atmospheric pressure.

Two essential components are required for plasma generation: a high-voltage source and a reactor [62]. A high-voltage source is used to establish a high electric field across the reactor. Key parameters affecting system efficacy relate to the form of the applied voltage (direct or alternating current, power, low or high frequency, pulsed or continuous operation, microwave, etc.) and the particular configuration of the reactor, depending on factors such as electrode arrangement, gas flow, and pressure. The most common systems in food applications are summarized below [63].

**Figure 1:**
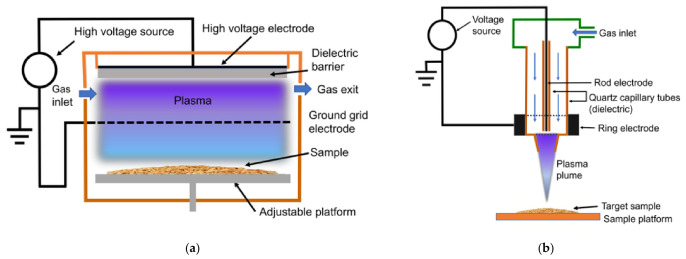
Schematics of a surface DBD plasma (**a**) and a cold plasma jet (**b**) system.

**Figure 2:**
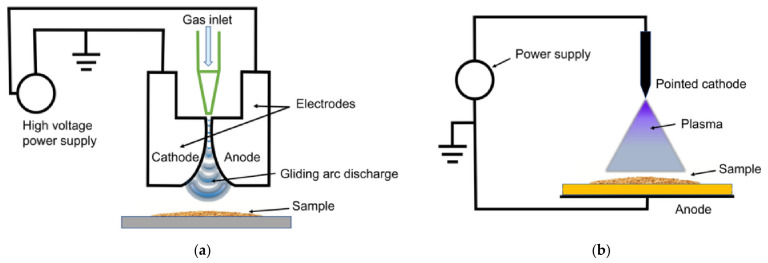
Schematics of a gliding arc discharge plasma (**a**) and a corona discharge plasma (**b**).

**Figure 3:**
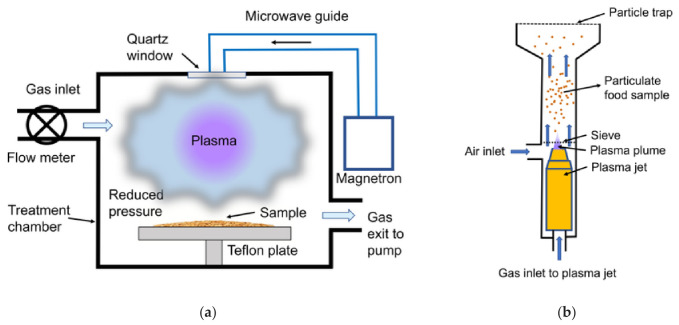
Schematic representation of a microwave discharge plasma (**a**) and a fluidized bed plasma reactor (**b**).

- Dielectric barrier discharge (DBD). The device comprises two electrodes. Usually, one is at high voltage, and the other is grounded, and they are separated by a dielectric material, such as glass, quartz, ceramic, or polymer. The dielectric barrier plays a crucial role in regulating the current flow, preventing the formation of an electric arc, and enabling the ionization of gas within the space between the electrodes (Figure 1a). The occurrence of a discharge within the volume occupied by a gas gap between two parallel electrodes or coaxial cylinders, with one or both electrodes covered by a dielectric material, is designated as a volume dielectric barrier discharge. This system is referred to as dielectric barrier discharge (DBD) or surface dielectric barrier discharge (SDBD), depending on the electrodes and dielectric barrier configurations employed. A mesh-electrode SDBD employs a mesh or grid ground electrode with the sample positioned directly beneath it or above it (Figure 1a). When a high voltage is applied to one of the electrodes while the other is grounded, the gas in the space experiences an increase in voltage and ionizes, forming a non-thermal plasma. The dielectric accumulates a charge that weakens the electric field, thereby triggering the discharge to extinguish. This process is repeated rapidly in each voltage half-cycle, resulting in the formation of numerous short-lived, pulsed plasma filaments. The distance between the electrodes varies depending on the particular application. In the SDBD configuration, both electrodes are in direct contact with the dielectric material, thereby eliminating the need for a gap. Additionally, the electrodes are laterally shifted from each other, a feature that enables asymmetry. The plasma is created on the dielectric material adjacent to the exposed electrode. Gases, including air, N_2_, Ar, and He, can usually be utilized at atmospheric pressure. Diffuse coplanar surface barrier discharge (DCSBD) is a specific configuration of DBD that utilizes a coplanar electrode arrangement embedded in or on a dielectric surface, in contrast to the conventional volume DBD, where a thin, flat, and apparently homogeneous plasma is generated between two larger plate electrodes. It is one of the most prevalent CP devices employed in the decontamination of fungi and mycotoxins in foods [5,7,42,46,55,63].

- Plasma jet. This plasma type, also known as a non-equilibrium atmospheric-pressure plasma jet (N-APPJ or APPJ), is a non-thermal plasma cylindrical device in which a partially ionized gas is generated within a small discharge chamber and then expelled by a gas flow as a directed plume into the surrounding atmosphere. Gas flow is a key variable for applications. The discharge is produced by applying high-voltage, either radio-frequency, pulsed, or microwave, to a flowing feed gas such as He, Ar, or their mixtures with O_2_ or air. The high-voltage electrode is a ring placed over the tube, and a bar positioned in the middle of the cylinder acts as the ground electrode (Figure 1b). Jet plasma systems are available in various configurations. They are not continuous plasma glows. Instead, they are made of plasma bullets that propagate at very high velocities, up to 105 m/s [46,56]. Therefore, the inner ground electrode can be replaced by a second ring-shaped electrode located before the positive electrode. In this configuration, the electrodes are separated and insulated by a dielectric material to prevent arcing outside the tube [63]. From an application perspective, the primary distinction between DBDs and plasma jets lies in their confinement. DBD plasmas are confined to the inter-electrode gaps or the chamber while APPJs allow the release of ionized species beyond these limits [42].
- APPJs have proven to be an effective solution for the decontamination of mycotoxins [6]. Their operation at ambient temperature, precise targeting, and ability to treat irregular or heat-sensitive materials without substantial thermal damage make them a highly desirable technology. Atmospheric pressure capacitively coupled plasma (AP-CCP) is a process that employs capacitive coupling for excitation and is frequently utilized to generate plasma jets. The AP-CCP supplies the energy required for the creation of plasma, which subsequently flows out as a jet.
- Gliding arc discharge plasma. The gliding arc discharge (GAD) plasma is a type of non-thermal plasma generated at atmospheric pressure between two diverging electrodes of different polarities (Figure 2a). A gas flows between the electrodes, and when a high voltage is applied across the two closely spaced electrodes, an arc is initiated at the point of minimum gap (the shortest distance between the electrodes). As the feed gas, commonly air, O_2_, or N_2_, flows through the system, the arc is elongated and lifted toward the region of wider electrode spacing, where it eventually extinguishes when the power supply cannot sustain an arc anymore and re-ignites at the shortest gap between the electrodes, producing a continuous sequence of transient plasma events. Although the core of the arc reaches relatively high temperatures, the expanding plasma column rapidly cools, enabling the surrounding effluent to remain in the non-thermal regime suitable for treating heat-sensitive materials. It has been classified as a warm plasma because of its gas temperature between cold and thermal plasmas. Gliding arc systems are efficient generators of reactive oxygen and nitrogen species and are increasingly explored for the decontamination of food surfaces, suppression of fungal growth, and degradation of mycotoxins [47,64,65]. Their ability to produce high densities of chemically active species at low operational cost makes it a promising technology for scalable food-safety interventions.

- Corona discharge plasma. This is a type of non-thermal plasma produced when a high electric field is applied across an asymmetric electrode configuration. These devices feature one or more sharp needles, points, or thin wires facing a planar or cylindrical ground electrode. When a strong electric field is applied to the system, the free electrons are accelerated, initiating a cascade of ionization, excitation, and dissociation reactions in the surrounding gas. When the voltage exceeds the gas breakdown threshold, a weakly ionized plasma is created. These discharges are relatively low-power electrical discharges at or near atmospheric pressure. These devices involve the flow of electric arcs between positive and ground electrodes, which are typically needle-shaped. To increase the applied area of the CD plasma aiming at food applications, a multipoint-plate electrode configuration has been gaining prominence due to its capacity to produce an energetic and dense plasma, creating a diffusive discharge with much more extensive coverage of the sample surface than a pin tip (Figure 2b). CD plasmas maintain electrons at high temperatures while the bulk gas remains near ambient temperature. This feature enables the treatment of heat-sensitive biological matrices including foods [66,67].
- Microwave discharge plasma. This type of plasma generates when a gas is exposed to microwave-frequency electromagnetic fields between 300 MHz and 300 GHz, most commonly at 2.45 GHz. It uses low voltage but high power (up to 1.2 kW). The microwaves, usually generated in a magnetron, travel through a waveguide into a chamber and supply energy to gas-free electrons, which collide with gas molecules (e.g., Ar, He, N_2_, O_2_, air) contained in the chamber, causing ionization and creating a self-sustaining plasma (Figure 3a). Generally, there are no electrodes, but there is a gas mass flow rate controller and a vacuum pump. The pressure inside the chamber is controlled by a vacuum valve and ranges from 500 to 30,000 Pa [68,69]. Microwave plasmas can also be powered by solid-state microwave generators, and it is possible to operate at atmospheric pressure. Microwave plasmas tend to be more uniform than DC or RF plasmas and can achieve high densities at low or atmospheric pressure, thus emerging as a powerful tool in the realm of mycotoxin decontamination.

- Radio frequency (RF) plasma. A high RF alternating voltage applied to the electrodes accelerates free electrons, causing them to collide with neutral gas molecules and finally produce the plasma. It is sometimes confused with MW-generated plasma, but typically works at 13.56 MHz or low frequencies (in the range of kHz). It requires high voltage, uses DBD systems, and needs electrodes to create the plasma. It can be operated as a capacitively coupled plasma (CCP), which uses a gas in the gap between two electrodes and is more usual in food processing, or inductively coupled plasma (ICP), which uses an RF current that circulates through a coil. This system can effectively inactivate aflatoxigenic fungi and degrade aflatoxins [4].
- Fluidized bed plasma reactor. This is a hybrid system that combines the principles of a fluidized bed with a plasma generation method, such as APPJ or DBD, inside a closed reactor vessel. The main feature is the fluidization of solid particles (e.g., kernels), using a gas flow, which allows for uniform mixing and high mass transfer rates between the plasma and the particle surface (Figure 3b). If the pressure is atmospheric, it is known as an atmospheric pressure fluidized bed plasma (APFBP) reactor. Therefore, food particles are suspended and agitated, behaving like a fluidized bed [2,70].
- Glow-discharge plasma. This system is generated when a gas is ionized inside a sealed chamber operating at low pressure, around 1 Torr or less. The chamber is first evacuated, and then a controlled flow of gas is introduced and exposed to high voltage applied between two electrodes. Under low-pressure conditions, the electric field accelerates free electrons, initiating collisions that produce ions, radicals, and additional electrons, thereby sustaining the plasma. A vacuum pump is responsible for maintaining a consistent low-pressure environment and removing the spent gases, thereby ensuring a uniform flow through the system. The power supply typically operates at RF or MW frequencies to sustain the discharge. High-frequency fields transfer energy to electrons, enabling ionization at low atmospheric pressures and producing the characteristic “glow” region where most plasma-chemical reactions occur [46,71,72].

The different CP devices are compared in Table 1.

## 3. Factors Influencing the Antifungal and Antimycotoxin Activity of CP

The generated CP interacts with the target surface in an indirect, semi-direct, or direct way [73]. In direct CP systems, the reactive species in the plasma discharge come into contact with the target surface. In this case, there is a highly effective interaction between the electrons, UV radiation, or short-lived radicals and the target, which promotes an efficient inactivation of microbiological species. However, sensitive targets may experience changes in their properties or be damaged [59]. In the semi-direct approach, a barrier, such as a mesh, is strategically placed between the plasma source and the target to reduce the treatment intensity, and in this post-discharge region, the long-lived particles are present, but not the short-lived ones. An indirect approach is employed when the target is exposed only to the “afterglow” or plasma-processed gas/liquid produced remotely from the plasma source. This approach is related to plasma-activated water (PAW) or plasma-treated liquid and limits the interaction of plasma reactive species with the target, thereby reducing the overall inactivation effectiveness.

The efficacy of these treatment methods has been thoroughly researched. The findings revealed that indirect plasma treatment is significantly less effective at disrupting bacterial biofilms formed by *E. coli*, *Bacillus* sp., and lactic acid bacteria [74].

A variety of factors influence the activity and efficacy of cold plasma in decontaminating and detoxifying food to reduce the presence of mycotoxins. The following are key points to consider:

- Type of device: DBD, plasma jet, corona discharge, microwave, among others, and their configurations.
- Type of exposition of targets to CP: direct, indirect, semi-direct.
- Type of target: class of food (e.g., cereal grains, nuts, spices, fruits, herbs, meat, etc.) and its appearance (e.g., ground, whole grain or nut, etc.), fungal species, type of mycotoxins (e.g., AFs, OTA, trichothecenes, ZEA, fumonisins, etc.).
- Gas used to create the plasma (O_2_, N_2_, air, humid air, noble gases (Ar, He), and their mixtures and applied pressure (atmospheric pressure, vacuum) or flow-rate.
- Applied voltage (which varies as a sinusoidal wave), frequency, power, distance between the cold plasma and the target, and treatment time.

The influence and the relevance of these variables on the effect of the CP treatment have been reported [46]. Due to the wide variety of influencing parameters, the information available is dispersed, which makes it difficult to obtain optimized CP equipment for use in the food industry to eliminate mycotoxins.

## 4. Mechanisms of Action of CP Against Toxigenic Fungi

As a result of extensive research, CP has demonstrated its capacity to inactivate toxigenic fungi at multiple sites. Fungi are multicellular filamentous molds constituted by cells that connect, forming hyphae. These hyphae result in long, branched filaments, which form an intricate network of threads denominated as the mycelium. In the fungal kingdom, certain cells are metamorphosed to serve a specialized function as conidiophores, giving rise to elongated stalks that culminate in swollen vesicles. These vesicles are the bearers of chains of asexual spores, scientifically termed conidia. These conidia constitute the reproductive system of the fungus. The structural organization of these organelles varies across different genera; however, they are consistently comprised of a protoplast that contains a nucleus, with the DNA enclosed within a membrane and an external cover that serves to protect the conidia against desiccation and damaging agents. The fungal cell is eukaryotic, meaning that it possesses a nucleus containing DNA, as well as a plasma membrane made of a double layer of phospholipids containing ergosterol units and transmembrane proteins. Other structures include the cytoplasm, Golgi apparatus, endoplasmic reticulum, mitochondria, and vacuoles. The membrane is covered by a cell wall composed primarily of the polysaccharide glucan, situated between an external layer of mannoprotein and an internal layer of the polysaccharide chitin. The stability of the cell wall is subject to constant enzymatic remodeling in response to proliferation and environmental challenges [39,75].

As previously indicated, multiple factors may influence the effectiveness of CP against fungi, including the type of plasma generator, working gas, processing parameters, differences among fungal species, and treated matrix.

Reactive oxygen and nitrogen species (RONS) have been identified as the primary molecules responsible for the antifungal effects of CP [46,62]. The generation of ROS and RNS radicals has been demonstrated to result in more effective inhibition by damaging fungal cell walls, leading to cell rupture and the leakage of intracellular fluids. Plasma exposure can cause the oxidation of lipids in cell membranes, resulting in a reduction in the cell membrane’s osmotic ability. Consequently, proteins, DNA, and enzymes within cell membranes are destroyed [76]. The highly reactive short-lived species •O and •OH from the direct CP react with components of the cell wall and cell membrane (lipids, proteins, and polysaccharides), where the double bonds of the lipids are rapidly peroxidized.

**Figure 4:**
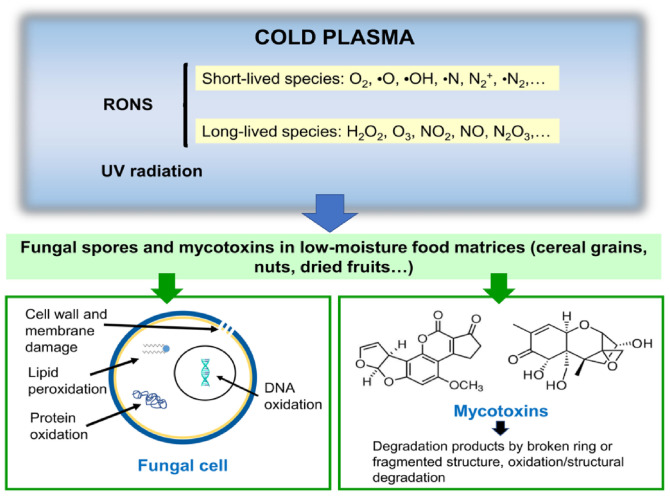
Schema of the interaction of cold plasma with fungal spores and mycotoxins in contaminated nuts, grains, or dried fruits.

The activity of RONS on fungal spores and mycotoxins that can be present in low-moisture foods (nuts, cereal grains, or dried fruits) is schematized in Figure 4.

In direct configurations, the activity of the UV radiation is also relevant [73]. These ROS have the capacity to react with the cellular RNA and DNA, as well as other organelles within the cell, ultimately resulting in cell death.

In indirect CP configurations, other less reactive, long-lived species, such as O_3_ or H_2_O_2_, with a negligible contribution of short-lived species due to the distance between the plasma and the target, are primarily responsible for the deterioration of the fungal cells’ structures, including the conidia, which exhibit higher resistance compared to the hyphae.

Zhao, X. et al. [77] investigated the in vitro effect of a DBDP device on the destructive effects and mechanisms of different voltage treatments on AF-producing *A. flavus* strains. The inactivation curve was fitted using Weibull and Logistic kinetic models. The results of the viable spore count indicated that following six minutes of 60 V treatment, the total number of viable spores diminished by 4.47 log CFU/mL. The leakage of nucleic acids and proteins, the level of lipid peroxidation, the reactive oxygen level, and the FTIR spectroscopy analysis of conidia confirmed that the cell membrane of *A. flavus* was severely damaged. The primary antifungal mechanisms of high-pressure CP include lipid peroxidation, protein oxidation, and DNA oxidation (Figure 4). Lipid peroxidation is attributed to the susceptibility of unsaturated fatty acids to attacks by hydroxyl radicals. Protein oxidation is the result of the susceptibility of amino acids to oxidation. DNA oxidation is the result of the formation of base adducts, which are generated through reactions with plasma radicals [2]. Morphological alterations of the fungal cells, such as conidia aggregation in clusters, phialide damage, cell shape changes, disruption of conidia sheaths, or cracks and pore-like structures on the conidial surface, have been observed in *Aspergillus* spp. [2,4,70,78]. Wang et al. [79] revealed that in *F. graminearum*, smaller-sized spores exhibited heightened susceptibility to reactive species due to their augmented specific surface area, consequently resulting in enhanced inactivation efficacy. Furthermore, spore germination rates diminished to less than 10% following 2 min of exposure to CP generated with a dielectric barrier surface microdischarge (SMD). The antifungal effects of CP against *F. graminearum* were mainly attributed to membrane destruction, intracellular ROS generation, and mitochondrial membrane potential loss. The decontamination of food particulate products (e.g., grains, nuts) by CP poses a significant challenge. This is likely attributable to the irregular surface structure and geometry of the food products. The microorganisms are protected by the uneven surface, small particles, or are hidden deep inside the surface, rendering them inaccessible to the decontamination process [80]. It is acknowledged that the process of inactivating *F. graminearum* conidia on wheat kernels (in vivo) is significantly more challenging than in water (in vitro). This is because fungal conidia located at the bottom of the grains are not effectively inactivated. Additionally, the conidia can be covered by the pappus and become embedded within the fissures of the kernels [79]. The combined effect of these factors protects the conidia from the attack of RONS.

## 5. Application of CP to the Reduction of Toxigenic Fungi in Food

Two methodologies exist for the elimination or reduction in mycotoxins in food using CP. On the one hand, as previously indicated, CP has antifungal activity, being capable of destroying fungal structures, which has been demonstrated in many studies. The elimination of toxigenic fungi in food is a critical step in preventing the production of mycotoxins. Furthermore, CP has been observed to impede mycotoxin biosynthesis by modulating the expression of genes associated with toxin production. The chemical species are directed to induce oxidative stress on the outer layers of the target conidia. The oxidative stress further disrupts the metabolic events of the cell, resulting in a disturbance of the cell membrane and its essential components, including DNA, proteins, and lipids (Figure 4). This, in turn, leads to a decline in the growth of the fungus and a reduction in the production of toxins [7]. For instance, the exposure of *A. ochraceus* to CAP resulted in ruptures and desiccations in fungal spores, accompanied by a substantial decline in their viability [78]. An alternative approach involves the direct action of the CP-generated reactive species on existing mycotoxin molecules. This process results in the degradation of the mycotoxin molecules into other, less toxic products.

### 5.1. Inactivation of Toxigenic Fungi in Nuts and Dried Fruits

As summarized in Table 2, studies differ greatly in terms of plasma systems, operating parameters, and food matrices, which complicates a direct comparison of their results. Reported reductions generally range from 2 to 6 log CFU/g; however, this variability not only reflects differences in treatment conditions such as power, voltage, gas composition, and exposure time, but also the influence of the matrix itself.

(a) Pistachios

Pistachios represent one of the most extensively studied matrices and therefore provide a useful case for comparing plasma configurations and treatment efficacy. For example, several plasma systems, including atmospheric pressure capacitive coupled plasma (AP-CCP), direct current diode plasma (DC-DP), and inductively coupled plasma (ICP), were compared using argon as the working gas [81,82]. AP-CCP achieved the highest efficacy among these systems, reaching complete inactivation (approx. 6 log CFU/g) at 150 W in 10 min, whereas DC-DP required higher power (300 W) and longer treatment times (20 min) to achieve slightly lower reductions (about 5 log CFU/g). ICP showed limited efficacy (2 log CFU/g) under the tested conditions. These differences likely reflect differences in reactive species generation and plasma density across the configurations. However, the study also reported minor alterations in pistachio shells, indicating a trade-off between microbial inactivation and product quality.

Likewise, DBD systems have demonstrated rapid inactivation kinetics. As listed in Table 2, Makari et al. [83] reported up to 4 log CFU/g reduction and complete inactivation within 3 min using ambient air, suggesting that shorter treatments may be effective under high-voltage conditions. In contrast, Esmaeili et al. [7] observed that longer exposure times (up to 15 min) and specific gas mixtures (Ar/air) were required to maximize fungal reduction, with voltage identified as a key influencing parameter. These discrepancies underline the strong dependence of treatment efficacy on both plasma parameters (e.g., voltage, power, gas composition) and experimental design.

Generally, while high levels of *A. flavus* inactivation can be achieved in pistachios, the absence of standardized CP conditions and the wide range of operating parameters limit both reproducibility and cross-study comparability. In addition, few studies have systematically evaluated quality changes or scalability. This poses serious challenges for industrial applications.

(b) Hazelnuts

CP treatments applied to hazelnuts (Table 2) showed significant antifungal efficacy against *A. flavus* and *A. parasiticus*, with described reductions usually ranging from about 2 to >5 log CFU/g depending on the CP configuration and working conditions. Nevertheless, as observed across studies, the efficacy of treatment is strongly influenced by the type of CP system, composition of feeding gas, and exposure time.

For instance, the atmospheric pressure fluidized bed plasma (APFBP) system investigated by Dasan et al. [70,80] accomplished reductions in the range of 4.2–4.5 log CFU/g after 5 min of treatment using dry air at high voltage (10 kV). These studies also showed that increasing voltage and frequency improved fungal inactivation, emphasizing the importance of energy input in CP efficiency. Likewise, Sen et al. [85] compared atmospheric pressure (AP-CP) and low-pressure (LP-CP) systems and reported similar or slightly higher reductions (4.4–5.6 log CFU/g), with air generally working better than N_2_ as a feeding gas, which suggests a key role of ROS in fungal inactivation.

The influence of gas composition was confirmed by Mošovská et al. [84], who proved that O_2_ and humidified synthetic air caused a complete or near-complete inactivation of *A. flavus*, whereas N_2_-based plasmas were considerably less effective. The enhanced efficacy in O_2_-containing CP systems was attributed to higher O_3_ production, indicating that specific reactive species are critical to attaining good antifungal activity.

Overall, as in the case of pistachios, the variability in CP sources and operating settings (Table 2) limits the comparison among studies. Moreover, few studies have systematically addressed the impact of treatment on product quality or the scalability of fluidized or low-pressure systems.

(c) Walnuts

Compared to hazelnuts and pistachios, few studies have evaluated CP for fungal inactivation in walnuts (Table 2), thus limiting cross-study comparison. Available data indicate that the complete inactivation of *A. flavus* can be achieved using plasma jets under relatively short treatment times (10–11 min) with Ar as the working gas [86]. Similar inactivation times were observed for both fresh and dried walnuts, which suggests that moisture content may have a limited influence under these conditions. However, the lack of variation in CP parameters and the scarcity of studies make it difficult to generalize these findings or assess reproducibility.

(d) Peanuts

The application of CP to peanuts (Table 2) has been more extensively studied. The reported antifungal effects range from partial inhibition (about 2 log reduction) to complete inactivation, contingent on the treatment conditions. As in other matrices, power, exposure time, and composition of the feeding gas are basic factors influencing the efficacy.

For example, treatments at moderate power (60 W) using low-pressure, capacitively RF-coupled glow discharge air plasma resulted in high inhibition rates (>97%) of *A. parasiticus* and *A. flavus* [4], while high-power plasma jets (180–200 W) reached reductions of more than 4–5 log CFU/g in a few minutes [87]. These results indicate a clear correlation between power input and inactivation effectiveness. Also, DBD systems have shown effective reductions (about 3.5 log) under high voltages, with negligible impact on organoleptic properties [88], underlying their capacity for practical applications.

Gas composition plays a critical role. The use of Ar/O_2_ mixtures [76] and air-based systems usually increases antifungal activity, likely due to the generation of ROS. Mechanistically, plasma-induced damage to fungal spores has been linked to membrane disruption via electroporation and surface etching [4], although such observations are often based on limited experimental evidence.

Despite these results, a substantial variability exists across studies in terms of treatment conditions and attained results (Table 2). Moreover, even when some studies addressed quality attributes, assessments of reproducibility, scalability, and long-term effects continue to be limited, underscoring the need for more standardized and application-oriented research.

(e) Roasted coffee beans and dried fruits

CP applications to coffee beans and dried fruits (Table 2) show antifungal potential, but the efficacy differs depending on both the matrix and CP conditions. For example, DBD treatment of roasted coffee beans contaminated with several *Aspergillus* species (*A. niger*, *A. westerdijikiae*, *A. steynii*, and *A. versicolor*) occasioned similar reductions (about 4 log CFU/0.1 g) across all species after 6 min under He/air plasma conditions [89]. This fairly uniform response suggests that under controlled conditions, species-specific differences may be less critical than the plasma parameters, although such conclusions are limited by the use of a single experimental work.

On the other hand, studies on dried fruits indicate greater variability in inactivation efficacy, thus reflecting differences in surface structure, composition, and matrix porosity. Dried fruits are generally susceptible to mycotoxin contamination due to production in warm climates, with figs reported as one of the most challenging matrices [91]. For instance, plasma jet treatments of dates achieved the complete inactivation of *A. niger* within 9 min, although lower reductions (around 1.7 log CFU/mm^2^) were observed at shorter treatment times [90]. In the same way, more limited reductions (1.5 log CFU/g after 12 min) were reported for dried figs under similar high-voltage CP conditions [92]. These differences emphasize the strong influence of the food matrix on CP efficacy, as more complex surfaces possibly limit the interaction between reactive species and fungal cells. While CP demonstrates clear capacity for the decontamination of coffee and dried fruits, the variability in treatment results underscores the need for a better understanding of matrix effects, as well as standardized processing conditions to enable a reliable comparison.

### 5.2. Inactivation of Toxigenic Fungi in Cereal Grains

Cereal grains are among the commodities most susceptible to contamination by toxigenic fungi during both pre- and postharvest stages. As summarized in Table 3, CP has usually been investigated as an approach for fungal decontamination in these matrices, but reported efficacy varies considerably across studies due to differences in plasma systems, operating parameters, and grain characteristics, which complicates a direct comparison of results.

(a) Barley grains

Studies on barley grains (Table 3) indicate that CP treatments generally lead to moderate reductions in fungal contamination, usually below 3 log CFU/g. With DCSBD, reductions of *A. niger* and *P. verrucosum* around 2.5–3 log cycles have been reported after two weeks, particularly when dry air is used instead of CO_2_-based mixtures [93]. In contrast, the gliding arc discharge (GAD) plasma jet appears less effective, with reductions of roughly 1 log CFU/g even after relatively long treatment times (20–30 min) [65]. These observations suggest that both the plasma configuration and the working gas influence the outcome. In general, the effect of CP in barley seems more limited than in other matrices, indicating that further optimization is needed.

(b) Maize grains

Maize has been more widely studied than other cereal grains (Table 3), and in general, higher levels of fungal inactivation have been reported, typically in the range of 3–4 log CFU/g. In some cases, DCSBD treatments have produced reductions above 4 log CFU/g for *A. flavus* and *Fusarium* spp. when exposure times are optimized [94]. In contrast, results obtained with DBD systems tend to be more variable, usually falling between roughly 1 and 3 log CFU/g depending on factors such as frequency and treatment duration [7]. There is also evidence that air-fed plasmas perform better than other gas compositions, particularly in limiting fungal growth and germination [55]. Some comparisons with other food matrices suggest that under similar treatment conditions, CP can be more effective in maize than in hazelnuts [2,70]. This difference is often attributed to structural factors, such as surface roughness, which may influence how reactive species interact with fungal cells. Even so, the wide variation in experimental setups and reporting (Table 3) makes it difficult to directly compare results across studies, and this continues to limit reproducibility.

(c) Wheat grains

CP treatments in wheat (Table 3) generally result in relatively low reductions when applied directly to grains. For example, DBD micro-discharge CP achieved only about 0.4 log CFU/g reduction after 3 min, whereas complete inactivation was observed under in vitro conditions [79]. This reveals the strong protective effect of the wheat grain matrix, which can limit the accessibility and reactivity of plasma-generated species. Similarly, gliding arc plasma treatments can decrease fungal growth rather than accomplish complete inactivation, as reflected by increased lag times and reduced growth rates for *A. flavus* and *A. parasiticus* [47]. These findings suggest that in wheat, CP can be considered as a growth control approach rather than a full decontamination method. As in other grains, increased electrical power and treatment time enhance efficacy, although practical limitations related to product quality and scalability need to be considered.

(d) Rice grains

Rice grains (Table 3) showed variable responses to CP treatment, with reductions ranging from partial to considerable inhibition depending on the conditions. For example, treatments with plasma jet using Ar achieved *A. flavus* reductions of roughly 4 log CFU/g after relatively long (20 min) exposure times [97], whereas DBD systems resulted in moderate decreases (65–80%) in spore formation across different fungal species [10]. The effect of the gas composition is particularly patent in rice, where mixtures containing O_2_ significantly enhanced antifungal efficacy. For example, near-complete inactivation of *A. parasiticus* (99.98%) was achieved at high voltage with O_2_-enriched gas mixtures [95]. These results emphasize the importance of ROS in fungal inactivation, although the wide range of conditions and endpoints (growth versus spore formation) complicates direct comparison among studies.

(e) Other grains

Studies performed on other grains, including rice derivatives and buckwheat (Table 3), highlight the variability of CP efficacy concerning fungal species and matrices. For example, transformer discharge plasma showed species-dependent sensitivity, so that *A. fumigatus* was more susceptible than *Fusarium* spp., which exhibited only limited reductions in the range 1–1.9 log CFU/g under similar conditions [8]. On the other hand, low-pressure ICP treatments using O_2_ as the feeding gas provided rapid and extensive inactivation across several fungal species, achieving total or near-complete reductions in short exposure times (about 2 min) [96], which indicates that the choice of CP system and the fungal species significantly influence the outcomes.

As observed throughout this section, the lack of standardized methodologies and the diversity of experimental designs (Table 3) remain crucial challenges for comparing results and assessing the practical applicability of CP technologies in cereal grains.

## 6. Effects of CP on the Mycotoxins

The degradation of mycotoxins is related to a combination of diverse mechanisms, such as chemical reactions with reactive species and UV radiation generated by CP, which provide molecular changes by the treatment and produce other compounds whose toxicity is likely lower than that of the target mycotoxin [62]. Ten Bosch et al. [5] conducted a study on the degradation of various mycotoxins under the action of cold atmospheric pressure plasma, with ambient air chosen as the working gas. The mycotoxins identified in this study included DON, AAL toxin TA, sterigmatocystin, ZEA, enniatins, FB_1_, and T-2 toxin. Degradation rates exhibited variation in accordance with the mycotoxin structure: FB_1_ and the structurally related AAL toxin TA demonstrated the most rapid degradation, while sterigmatocystin exhibited the highest resistance to degradation. The degradation of all pure mycotoxins was nearly complete within 60 s; however, the matrix in which they were included increased the time required for degradation. The degradation kinetics of all pure toxins followed an exponential decay.

### 6.1. Degradation Mechanisms of Aflatoxins

**Figure 5:**
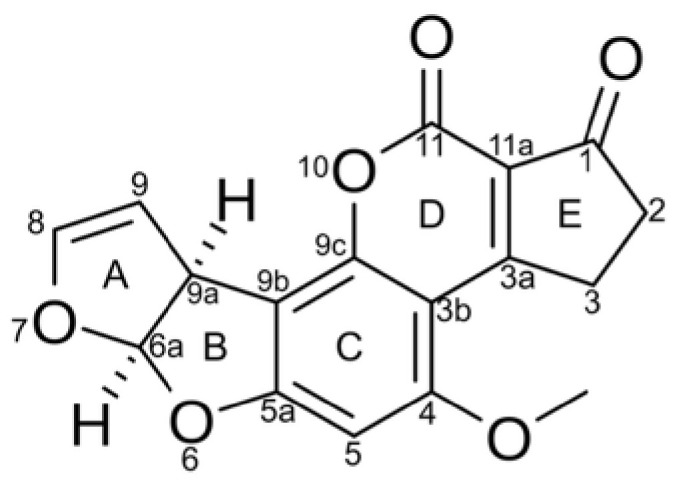
Structural formula of aflatoxin B_1_.

The mechanism of action of CP on the mycotoxins is evidently distinct for the various molecules. The most frequently reported effects of CP on mycotoxins are focused on AFs, particularly on AFB_1_ (C_17_H_12_O_6_), which has a molecular weight of 312.27. Its structural composition is characterized by the presence of a complex pentacyclic fused ring system. In this review, the five-ring segments of AFB_1_ are labeled A–E following the structural figure used in Martínez et al. [98], where rings A and B correspond to the fused furan units, ring C to the benzene-like moiety, with rings D and E as the lactone and cyclopentenone rings, respectively (Figure 5).

The numbering system employed in this formula aligns with the conventions established by Nicolás-Vázquez et al. [99] and Wang et al. [100]. The toxicity of AFB_1_ and AFG_1_ is primarily attributed to the C8=C9 double bond in the final furan ring [101]. The process of CP-reactive species disrupting this double bond results in the generation of less-toxic compounds [101,102]. The degradation rate of AFB_1_ by CP is contingent upon various factors, including gas type, power, voltage, and treatment time. Some studies have demonstrated substantial reductions, with percentages ranging from 55% in rice to 62.6% in mulberries and 68.59% in sesame seeds, among others. Consequently, CP is regarded as a promising non-thermal approach for food safety, although its impact on other components, such as lipids and phenolic compounds, and the extent of degradation vary depending on the food matrix.

**Figure 6:**
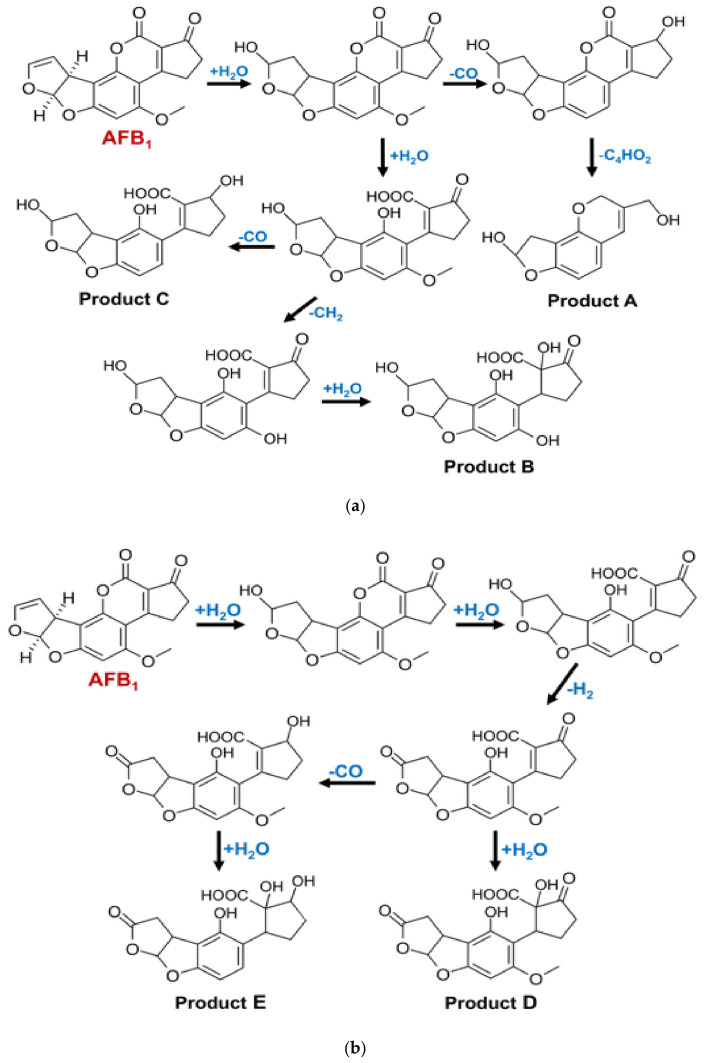
First (**a**) and second (**b**) pathways of AFB_1_ degradation by the activity of CP as suggested by Wang et al. [103].

Wang et al. [103] conducted a study on the degradation of AFB_1_ using low-temperature RF plasma. The study’s findings indicated that plasma could effectively degrade AFB_1_ (Figure 6).

Specifically, exposure to 300 W plasma for 10 min resulted in a degradation rate of up to 88.3%. The application of UPLC-Q-TOF-MS, equipped with an electrospray ionization source operating in positive ionization mode (ESI+), enabled the identification of up to five derived products, which were assigned the following formulas: product A (C_12_H_14_O_4_), product B (C_16_H_17_O_9_), product C (C_16_H_17_O_7_), product D (C_17_H_17_O_9_), and product E (C_16_H_17_O_8_). Two pathways leading to the degradation products are shown in Figure 6. It was observed that all of the products underwent a loss of the C8=C9 double bond in the terminal furan ring as a result of the addition of O and OH. This phenomenon may be attributed to the presence of reactive radicals •O and •OH. This finding suggests that the degradation products exhibited lower toxicity than AFB_1_, consistent with the structure–toxicity relationship hypothesis.

**Figure 7:**
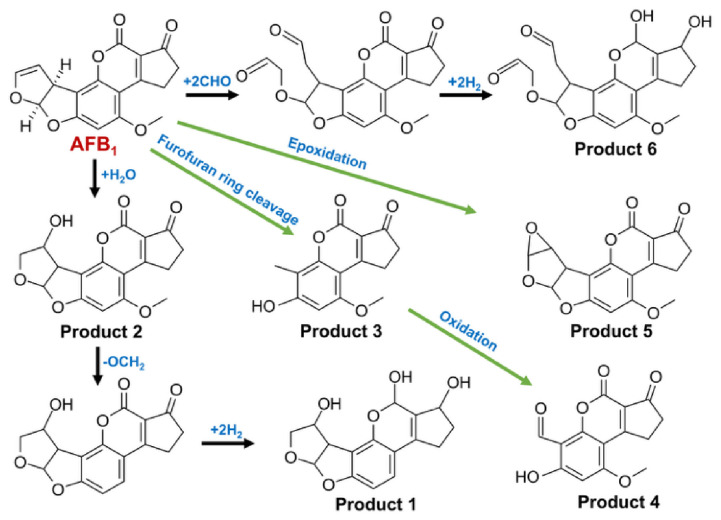
Degradation pathways of AFB_1_ by CP suggested by Shi et al. [104]. Pathway 1 follows the black arrows. Pathway 2 follows the green arrows.

Research conducted by Shi et al. [104] using high-voltage atmospheric cold plasma (HVACP) on AFB_1_ led to the finding of six degradation products from this mycotoxin. Their formulas, determined using LC-TOF-MS, are as follows: product 1 (C_16_H_16_O_6_), product 2 (C_17_H_14_O_7_), product 3 (C_14_H_12_O_5_), product 4 (C_14_H_10_O_6_), product 5 (C_17_H_12_O_7_), and product 6 (C_19_H_18_O_8_). The first two products were attributed to the effect of O_3_. The remaining products were attributed to O_3_ and other active species. All of the six proposed major degradation products of AFB_1_ lost the double bond at C8=C9, and the degradation products were different from AFB_1_ by further modification of the furofuran ring (products 1–6), lactone ring (products 1, 6), cyclopentenone (products 1, 6), or the methoxyl structure (product 1). Shi et al. [104] justified the pertinent pathways from AFB_1_ to these products, and such degradation pathways are shown in Figure 7. The six products are likely less toxic than AFB_1_. The authors expected that AFB_1_ degradation would occur through simultaneous reactions with ROS. Two different pathways were suggested. From the first degradation pathway, the key reactive agents were the hydrogen atom (H) and the •OH radical, which were generated by the breakdown of water molecules. These two species are responsible for hydration and hydrogenation to form new degradation products (products 1, 2, and 6). This first pathway involves an addition reaction in which a water molecule is added to the C8=C9 vinyl bond. Then, the methoxy group was cleaved, and the two carbonyl groups at the lactone and cyclopentenone rings were hydrogenated to give product 1. Also, the addition of the radical •CHO (due to the presence of CO_2_ in the gas) to the furan A ring renders a dialdehyde, and product 6 was the result of further hydrogenation of the carbonyl groups at the lactone and cyclopentenone rings. The second pathway involved an epoxidation reaction by hydroperoxyl radical and oxidation reactions through the combined effects of the oxidative species •OH, H_2_O_2_, and O_3,_ with cleavage of the furofuran ring. Products 3, 4, and 5 (AFB_1_-8,9 epoxide) were the result of this second pathway.

**Figure 8:**
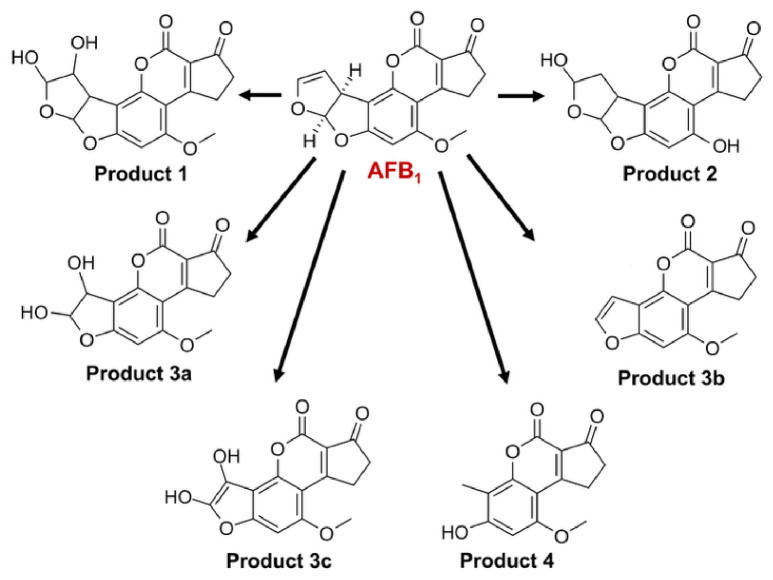
Degradation products of CP-treated AFB_1_ according to Wielogorska et al. [102].

Wielogorska et al. [102] demonstrated a decrease in AFB_1_ level, with up to six new compounds produced after 20 min of cold atmospheric pressure plasma (CAPP) jet treatment, with AFB_1_-dihydrodiol being one of these products. After analysis by HPLC-HRMS, their formulas were determined to be product 1 (C_17_H_14_O_8)_, product 2 (C_16_H_12_O_7_), product 3a (C_15_H_12_O_7_), product 3b (C_15_H_10_O_5_), product 3c (C_15_H_10_O_7_), and product 4 (C_14_H_12_O_5_) (Figure 8).

The structural identity of all cited degradation products by Wielogorska et al. [102], Wang et al. [103], and Shi et al. [104] is different among them. Therefore, the experimental conditions of the CP treatments of AFB_1_ seem to be very influential on the pathways followed by this toxin and the degradation products.

**Figure 9:**
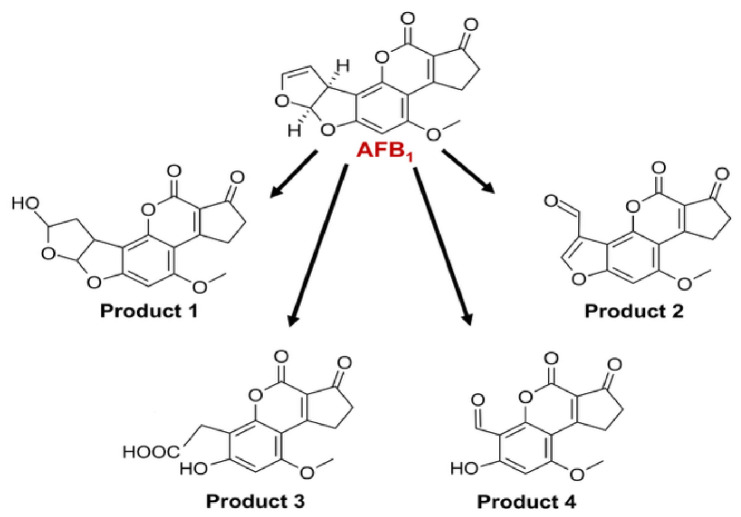
Structures of the main degradation products of AFB_1_ exposed to ACP according to Hojnic et al. [105].

Using high-resolution mass spectroscopy (HRMS) and nuclear magnetic resonance spectroscopy (NMR), Hojnic et al. [105] studied the degradation products of AFB_1_ after exposure to SBD plasma to provide a detailed description of CAP-mediated AFB_1_ degradation. They identified rapid scission of the vinyl bond C8=C9 on the terminal furan ring of AFB_1_ as being of paramount importance for the suppression of toxic potential, which was confirmed by the examination of cytotoxicity and genotoxicity. CAP-generated RONS must be the crucial agents responsible for AF degradation because thermal effects are negligible, and UV photons have much less effect on AFB_1_ degradation compared to RONS. Working with an initial level of 35 µg/mL, an AFB_1_ decontamination efficiency of 96% was achieved after only 60 s of treatment, and no AFB_1_ was detected after 120 s of exposure. The aflatoxins occurring in a food matrix are subjected to degradation by RONS generated by the CP, which break the chemical bonds of the molecules, changing them into other harmless products. These species interact with the AFB_1_ molecule, leading to chemical modifications such as oxidation, epoxidation, ring cleavage, and addition reactions. The four main degradation products of AFB_1_ found in vitro were product 1 (C_17_H_15_O_7_), product 2 (C_16_H_11_O_6_), product 3 (C_15_H_13_O_7_), and product 4 (C_14_H_11_O_6_), where the formulas correspond to the [M + H]^+^ ions and are depicted in Figure 9, where intermediate compounds included in the pathways are omitted [105] and formulas are those of the neutral molecules.

Product 1 is produced by the addition of OH and H to the C8 and C9 carbon atoms in the terminal furan ring of AFB_1,_ with the consequent break of the double bond (it is known as AFB_2a_). Product 2 was the result of the cleavage of the terminal furan ring and the formation of an aldehyde. Product 4 is the result of the cleavage of furan ring B by oxidation, with the formation of a carboxylic acid. Product 4 has an aldehyde group bonded to the benzene ring. Such structural changes finally reduce the AFB_1_ toxicity, making CP a promising technology for decontaminating food products.

**Figure 10:**
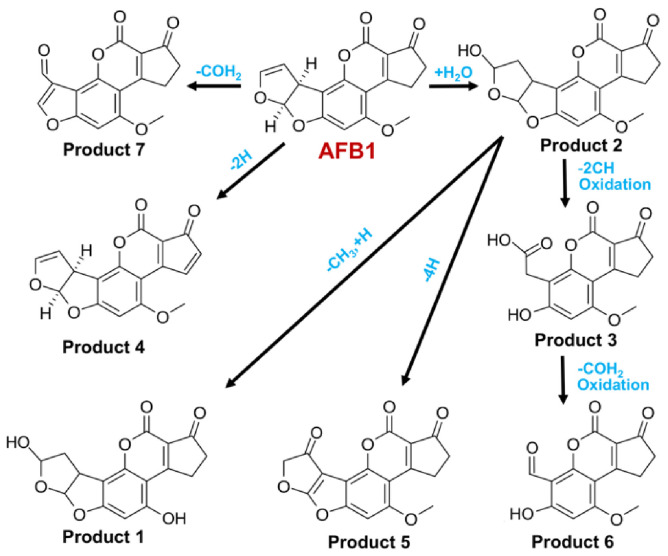
Degradation pathways of CP-treated AFB_1_ as suggested by Zhao et al. [106].

Zhao et al. [106] also proposed various degradation pathways for AFB_1_ exposed to DBD CP in glass slides. The products were characterized by Q-TOF-LC/MS/MS with the ESI set in the positive ion mode (ESI+), and the *m*/*z* values given correspond to the protonated [M + H]^+^ ions. Seven products were identified. The AFB_1_ molecule was destroyed by the disintegration of the furan ring, modification of the methoxy group, and formation of a double bond in the cyclopentenone cycle, thereby decreasing the toxicity of AFB_1_. The degradation pathways proposed are shown in Figure 10. Product 2 (C_17_H_15_O_7_, *m*/*z* = 331.0810) (identical to AFB_2a_) was the result of the addition of OH and H to the C8=C9 double bond of the terminal furan ring of AFB_1_. Product 1 (C_16_H_13_O_7_, *m*/*z* = 317.0656) was formed from product 2 by the change of the methoxy group to a hydroxyl and matches product 2 reported by Wielogorska et al. [102], taking into consideration the protonated ion. Product 5 (C_17_H_11_O_7_, *m*/*z* = 327.0501) was formed from product 2 by the creation of a ketone group at C9 and a double bond at the junction (C6a, C9a) of the two furan rings. Product 3 (C_15_H_13_O_7_, *m*/*z* = 305.0655) evolved from product 2 by opening the furofuran ring with the appearance of a carboxylic group and an OH group and matched product 3 of Hojnic et al. [105]. Product 6 (C_14_H_11_O_6_, *m*/*z* = 275.0546) was the result of the further oxidation of product 3, with the appearance of an aldehyde group, and agreed with product 4 of Shi et al. [104]. Product 7 (*m*/*z* = 299.0552) was the result of the destruction of the terminal furan ring (A) of AFB_1_ and the appearance of a double bond (C6a=C9a) in the furan ring (B); it matched product 2 suggested by Hojnic [105]; finally, product 4 (C_17_H_11_O_6_, *m*/*z* = 311.0531) evolved from AFB_1_ by the formation of a double bond between C2 and C3. The position of the OH group in C8 or C9 in products formed by the addition of OH and H to the C8=C9 double bond of AFB_1_ is different according to different articles, although the *m*/*z* values are identical (positional isomers).

Li et al. [107] employed reactive molecular dynamics simulation (RMD) with the reactive force field (ReaxFF) potential to computationally elucidate the possible intermediate radicals and molecules, as well as the pathways followed through AFB_1_ degradation by reaction with the ROS produced by CP. The results of the simulation indicated that ROS (O atoms, •OH, H_2_O_2_, and O_3_) can reduce the toxicity of AFB_1_ by the addition reaction of the double C8=C9 bond, the ring opening reaction of the terminal furan ring, and the destruction of the lactone ring. The reduction of the carbonyl group and double C=C bonds in the cyclopentenone ring can also be observed in the simulation. Coincidence of some products with those obtained experimentally by Wielogorska et al. [102] was claimed to validate the results of these theoretical studies.

**Figure 11:**
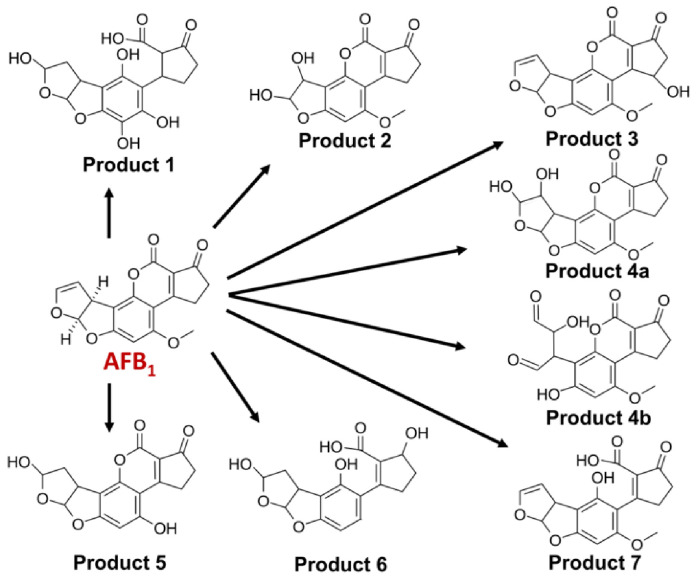
Degradation products of AFB_1_ in maize kernels treated with the gas effluent from a DBD plasma according to Somma et al. [55].

Somma et al. [55] investigated the degradation products of AFB_1_ in maize kernel samples contaminated with this toxin. The samples were exposed downstream to gas effluents from a DBD ACP system fed with O_2_ for 20 min. Seven degradation products were identified after accurate mass measurements by UHPLC-HRMS using the ESI in the positive mode, with an error lower than 2 ppm (Figure 11). The following formulas match the neutral molecules, but the *m*/*z* values are the exact masses of the protonated [M + H]^+^ ions. Product 2 (C_15_H_12_O_7_, *m*/*z* = 305.0626) was the most abundant and was the result of the cleavage of the terminal furan ring (A) and the dihydroxylation of the remaining furan cycle B. This product coincides with product 3a in Figure 8, in agreement with the results of Wielogorska et al. [102].

Somma et al. [55] identified product 3 (C_17_H_12_O_7_, *m*/*z* = 329.0656), which is related to AFQ_1_ or epi-AFQ_1_, a metabolic product of AFB_1_ produced by the cytochrome P450 enzyme that has an OH group at the cyclopentenone cycle (E). Product 1 (C_16_H_16_O_9_, *m*/*z* = 353.0867) resulted from the addition of an OH group to the benzene ring, the substitution of the methoxy group at C4 by an OH group, the addition of H_2_O to the C8=C9 double bond, the opening of the lactone ring (D), and the elimination of the double bond of the cyclopentenone ring. Product 4 (C_17_H_14_O_8_, *m*/*z* = 347.0761) had 34 Da more than AFB_1_, and was attributed to AFB_1_ 8,9-dihydrodiol (product 4a), which matched product 1 reported by Wielogorska et al. [102], or AFB_1_ 8,9-dialdehyde (product 4b), associated with the opening of the furofuran ring (A-B). Both molecules had the same molecular formula and the same *m*/*z* value. Product 6 (C_16_H_16_O_7_, *m*/*z* = 321.0969) was the result of the H_2_O addition reaction at the C8=C9 double bond, the opening of the lactone ring (D), the O-cleavage of the methoxy group at C4 of the benzene ring (C), and the reduction of the ketone group of the cyclopentenone ring (D) to produce an OH group. This product matched product C by Wang et al. [103] (see Figure 6). Product 5 (C_16_H_12_O_7_, *m*/*z* = 317.0656) was the result of a reaction between OH and H at the vinyl C8=C9 bond, as well as the substitution of the methoxy group at C4 with an OH group. The results of this study aligned with the degradation product 2 as reported by Wielogorska et al. [102] and product 1 as reported by Zhao et al. [106]. Product 7 (C_17_H_14_O_7_, *m*/*z* = 331.0812) is hypothesized to be derived from the lactone ring opening in the AFB_1_ molecule. A product with the same formula and *m*/*z* value was identified by Shi et al. [104], although both structures were different.

The summarized results of the research performed on the degradation products of AFB_1_ and their relationships are listed in Table 4.

### 6.2. Degradation Mechanisms of Ochratoxin A

**Figure 12:**
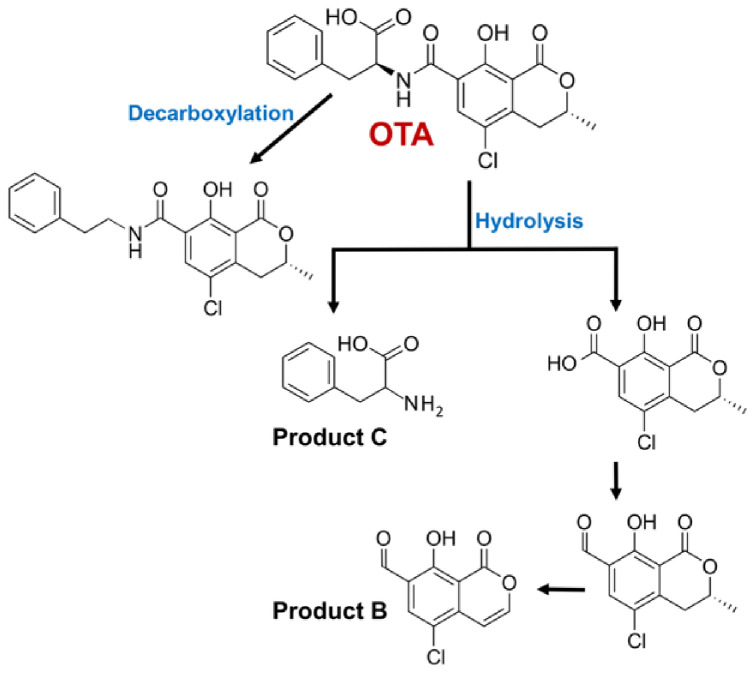
Degradation pathways of OTA exposed to CP proposed by Wang et al. [109].

Ochratoxin A (OTA) is a chlorinated isocoumarin derivative with a molecular formula C_20_H_18_ClNO_6_ consisting of a para-chlorophenolic dihydroisocoumarin moiety linked to the amino acid L-phenylalanine. CP can degrade OTA in different food commodities [10,40,89]. It is known that O_3_ can react with chlorinated molecules, such as OTA, producing free chlorine and amino acids [108]. The degradation activity of O_3_ on OTA is higher than that of NOx molecules [40]. In 2024, Wang et al. [109] reported the fate of OTA on exposure to a surface discharge plasma system fed with air. The analysis of the treated solution by HPLC-MS/MS found two degradation products (products B and C) that appeared after 3 min of treatment, while the peak of OTA gradually disappeared with increasing treatment time and was undetectable after 10 min. Product B also decreased with time and disappeared, while the peak of product C increased with time. Product B was considered an intermediate degradation compound. Further analysis by Orbitrap ion trap MS identified product B and product C. This last was assigned to L-β-phenylalanine based on an ion at *m*/*z* 158.1540 (Figure 12). However, some inconsistencies in molecular assignments were found in the report.

**Table 4:** Relationships among the CP degradation products of AFB_1_ as proposed by different studies.

Degradation Products of AFB_1_								CP System	Conditions	Ref.
Product A C_12_H_13_O_4_	Product B C_16_H_16_O_9_	Product C C_16_H_16_O_7_ ^(g)^	Product D C_17_H_16_O_9_	Product E C_16_H_16_O_8_				Low-temperature RF plasma	Press. 15 Pa; 300 W; 10 min	[103]
Product 1 C_16_H_16_O_6_	Product 2 C_17_H_14_O_7_	Product 3 C_14_H_12_O_5_	Product 4 C_14_H_10_O_6_	Product 5 C_17_H_12_O_7_	Product 6 C_19_H_18_O_8_			HVACP (DBD)	Air; 90 kV; 200 W; 50 Hz	[104]
Product 1 C_17_H_14_O_8_ ^(f)^	Product 2 C_16_H_12_O_7_ ^(b)^	Product 3a C_15_H_12_O_7_ ^(e)^	Product 3b C_15_H_10_O_5_	Product 3c C_15_H_10_O_7_	Product 4 C_14_H_12_O_5_			DBD plasma jet	He + 0.5–0.75% O_2_; 6 kV; 20 kHz	[102]
Product 1 C_17_H_14_O_7_ ^(a)^	Product 2 C_16_H_10_O_6_ ^(h)^	Product 3 C_15_H_12_O_7_ ^(c)^	Product 4 C_14_H_1o_O_6_ ^(d)^					SBD	Air	[105]
Product 1 C_16_H_12_O_7_ ^(b)^	Product 2 C_17_H_14_O_7_ ^(a)^	Product 3 C_15_H_12_O_7_ ^(c)^	Product 4 C_17_H_10_O_6_	Product 5 C_17_H_10_O_7_	Product 6 C_14_H_10_O_6_ ^(d)^	Product 7 C_16_H_10_O_6_ ^(h)^		DBD	Air; 160 kV; 50 Hz	[106]
Product 1 C_16_H_16_O_9_	Product 2 C_15_H_12_O_7_ ^(e)^	Product 3 C_17_H_12_O_7_	Product 4a C_17_H_14_O_8_ ^(f)^	Product 4b C_17_H_14_O_8_	Product 5 C_16_H_12_O_7_	Product 6 C_16_H_16_O_7_ ^(g)^	Product 7 C_17_H_14_O_7_	DBD	O_2;_ press. 760 Torr (indirect); 13.8 kV; 6 kHz; 20 min	[55]

All molecular formulas correspond to neutral molecules for easy comparison. Coincidences of molecular structures are noted by the same superscript letter (a–h). Coincidence of molecular formulas does not mean coincidence of structures.

**Figure 13:**
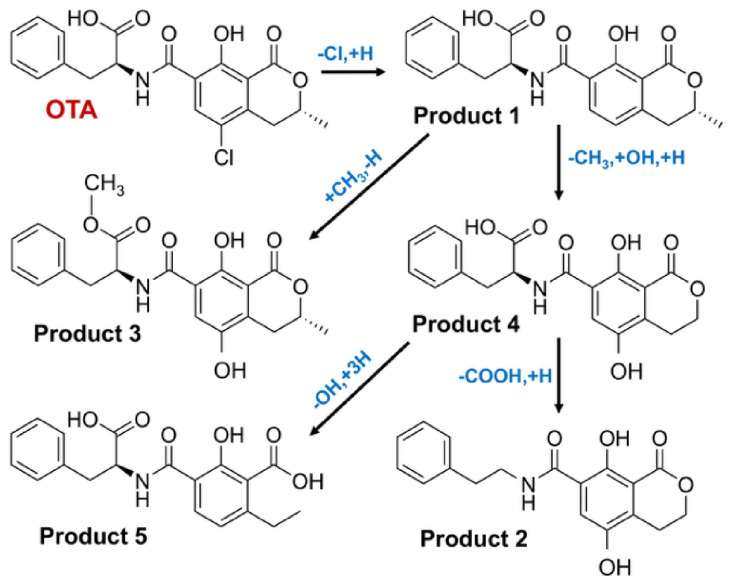
Degradation pathways of OTA by CP exposure according to Zheng et al. [110].

Zheng et al. [110] investigated the degradation products of OTA using a DBD-CP system (58 kV discharge voltage, 254 Hz treatment frequency, and 3.60 min treatment time). Using UPLC-Q-Exactive MS/MS with the ESI configured in negative ion mode (ESI-), they found five OTA degradation products through various possible reaction pathways, including dechlorination, hydroxylation/dehydroxylation, demethylation, esterification, decarboxylation, and lactone opening. The degradation products and their formulas, working with ESI-, were: product 1 (C_19_H_17_NO_7_), product 2 (C_18_H_17_NO_5_), product 3 (C_21_H_21_NO_7_), product 4 (C_20_H_19_NO_6_), and product 5 (C_19_H_19_NO_6_) (Figure 13). The proposed pathways are: (a) loss of the Cl atom (dechlorination) of OTA, which is changed by a hydrogen to provide product 4, whose formula is consistent with that of ochratoxin B (OTB), and was the main degradation product formed as observed in the chromatograms; (b) hydroxylation and demethylation of product 4 to render product 1; (c) hydroxylation and esterification of product 4 to give product 3; (d) loss of the COOH group of product 1 substituted by a hydrogen to give product 2; and (e) opening of the lactone ring of product 1 and elimination of the attached OH group through pathway c to produce product 5. The RONS formed during the treatment are likely responsible for the generation of these products rather than the heat or UV light produced by CP, which do not have sufficient energy to produce these modifications of the OTA structure [110].

### 6.3. Mechanisms of Degradation of Trichothecenes

**Figure 14:**
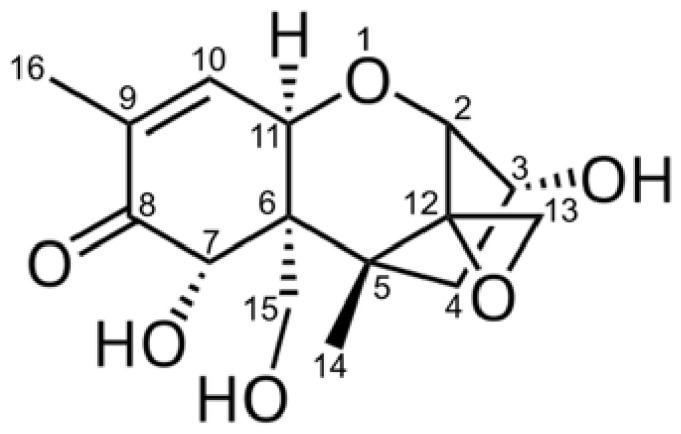
Structural formula of deoxynivalenol (DON).

The most important trichothecenes are type A and type B trichothecenes. All of them are susceptible to CP degradation. DON is the most relevant mycotoxin of the type B-trichothecenes. It is an epoxy-sesquiterpeneoid polar compound with the formula C_15_H_20_O_6_ and consists of a cyclohexene ring fused to a tetrahydropyranyl ring, which is further bridged to form a cyclopentyl ring (Figure 14). The structure has a C12,13-epoxide group (essential for toxicity), a double bond at C9=C10, and a ketone group at C8 in the cyclohexene ring, which defines it as a type-B trichothecene. The high oxidative potential of O_3_ on the DON molecule has been demonstrated [111]. The Criegee reaction mechanism for the cleavage of the double bond C9=C10 and the opening of the cyclohexene ring to provide a ketone/aldehyde degradation product was suggested. The oxidative process of O_3_ on this product continued, rendering degradation products, and leading to the opening of the C12,13-epoxy ring, providing a methylene group, and the further cleavage of the C2,3 bond with the subsequent transformation of C3 into a carboxylic group. Finally, the cleavage of the oxane ring at positions O1-C11 renders a fully oxidized acyclic degradation product [111].

**Figure 15:**
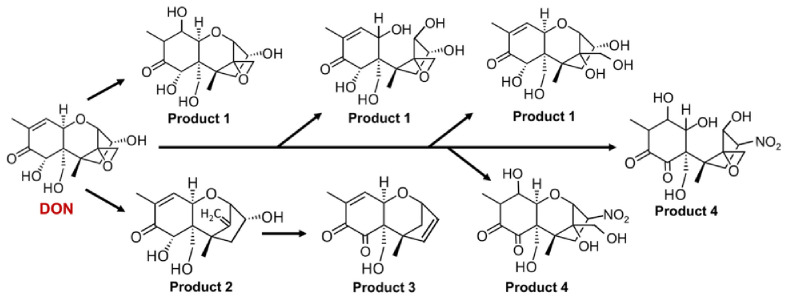
Possible degradation products and their pathways of CP-treated DON as proposed by Chen et al. [48].

Chen et al. [48] investigated the degradation products of DON by a DBD CP system using LC-TOF-MS for analysis of the degradation products. The key toxicity-related groups are the double bond at C9=C10, the C12,13-epoxy ring, and the OH linked to carbon C3 [112]. Then, the degradation products missing one or more of these groups should likely be less toxic than DON. Four molecular formulas of the possible degradation products of DON were obtained by screening the precursor ions: products 1 (C_15_H_22_O_7_), 2 (C_15_H_20_O_5_), 3 (C_14_H_16_O_4_), and 4 (C_15_H_21_NO_9_). The possible degradation pathways suggested by Chen et al. [48] are shown in Figure 15. Product 1 contained one oxygen atom and two hydrogen atoms more than DON; it might be formed by the addition of a molecule of H_2_O to the unsaturated bond (C9=C10) on the DON molecule. Water might also react with the C12,13-epoxy ring or with the cyclic ether of DON to form two OH groups each. Therefore, product 1 might have three potential molecular structures (Figure 15). Product 2 had one oxygen atom less than the DON molecule, which might be caused by the deoxygenation of the C12,13-epoxy group with the formation of a double bond [112]. Product 3 might be formed by the further degradation of product 2, losing one carbon atom, four hydrogen atoms, and one oxygen atom. Product 4 added one nitrogen atom, one hydrogen atom, and three oxygen atoms. The OH group at position C3 can be replaced by a nitro group, and the OH group at the C7 position can be oxidized, forming a ketone. The introduction of another water molecule may occur either at the 1-oxy group or at the C12 position. Then, according to the authors, product 4 might have two potential molecular structures. More research is needed to ensure the right structures of these degradation products.

The degradation products of DON dissolved in acetonitrile/water (20:80 *v*/*v*) and exposed to the activity of a DBD ACP system have been studied [113]. Different peaks from those of DON were observed in chromatograms, and their molecular formulas and *m*/*z* values were determined, although the structural formulas were not published. It was considered possible that during the ACP treatment, the OH group bonded to C3 could be oxidized to a ketone group, resulting in the reduced toxicity of DON.

**Figure 16:**
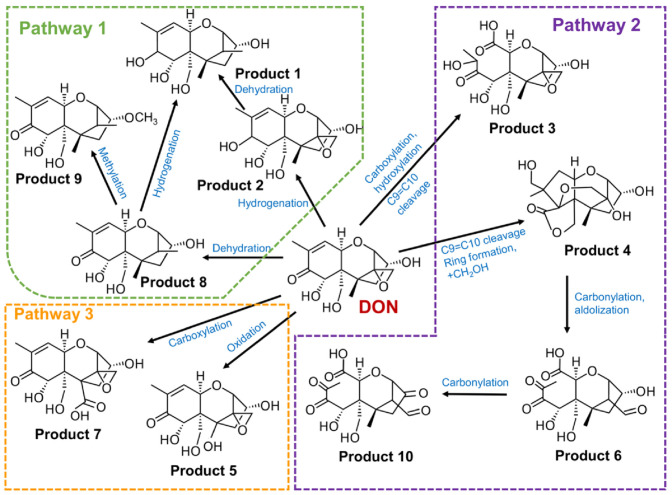
Degradation pathways of DON by ACP as proposed by Zhang et al. [114].

Ten degradation products of DON after being exposed to double dielectric barrier discharge (DDBD) cold plasma were detected by Zhang et al. [114] using UPLC-TOF-MS/MS analysis (with ESI-) of this mycotoxin. The degradation rate increased from 73.33% to 100% when the voltage increased from 60 V to 140 V, and also with increasing frequency. The molecular formulas of products 1 to 10 were C_15_H_24_O_5_, C_15_H_22_O_6_, C_15_H_22_O_9_, C_16_H_22_O_7_, C_15_H_20_O_7_, C_15_H_20_O_9_, C_15_H_18_O_8_, C_15_H_22_O_5_, C_16_H_24_O_5_, and C_15_H_18_O_9_, respectively. The proposed structures and three pathways suggested for DON degradation are shown in Figure 16.

In pathway 1, the C12,13-epoxy group was destroyed. Product 8 was generated through a dehydration reaction. It was converted to product 9 and product 1 by the methylation of the 3-OH group and the hydrogenation of the 8-carbonyl group, respectively. DON was transformed to product 2 by hydrogenation in the 8-carbonyl group. Product 2 was converted to product 1 through the dehydration reaction at the C12,13-epoxy group. Through pathway 2, the C9=C10 double bond of DON was cleaved, resulting in product 3, followed by conversion to product 6 via the carbonylation reaction of the 9-OH group and the elimination of the C12,13-epoxy structure with the generation of an aldehyde. Then, product 6 was converted to product 10 through the carbonylation reaction at the 3-OH group. Other reactions of DON involve the cleavage of the C9=C10 double bond, resulting in an addition reaction to form a CH_2_OH group, while the 7-OH and the 15-OH groups were destroyed and connected to the 8-carbonyl group into rings; in addition, the epoxy structure is hydroxylated to form product 4. In pathway 3, DON is converted either into product 5 by oxidation of the 5-methyl group or into product 7 by carboxylation of the 5-methyl group. Most of the products lack either the C9=C10 double bond or the epoxide structure. Thus, based on the relationship between structure and toxicity, it can be inferred that the toxicity of CP-treated DON decreases significantly [114].

**Figure 17:**
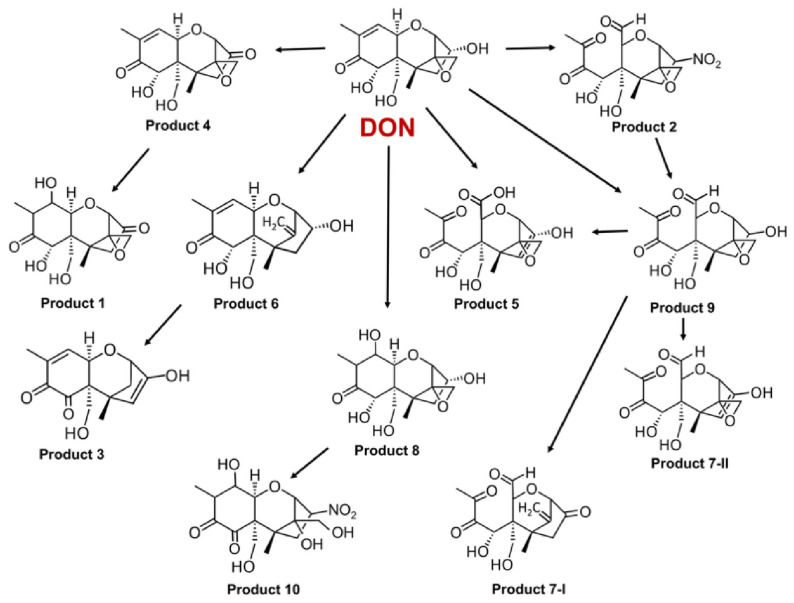
Degradation pathways of DON by ACP in solid and liquid phases as suggested by Fan et al. [115]. Products 1 and 2 were generated from DON only in the solid phase; products 7 to 10 were produced only in the liquid phase; products 3, 4, 5, and 6 were identified in both phases.

Fan et al. [115] compared the degradation efficiency of CAP against DON in the solid phase, aqueous solution, and wheat grains and formulated their degradation products and their pathways (Figure 17).

After ACP treatment, they identified six degradation products of solid DON: product 1 (C_15_H_20_O_7_), product 2 (C_15_H_19_NO_9_), product 3 (C_14_H_16_O_4_), product 4, also known as 3-keto-DON (C_15_H_18_O_6_), product 5 (C_15_H_18_O_8_), and product 6 (C_15_H_20_O_5_) as well as eight degradation products of liquid DON (product 3, product 4, product 5, product 6, product 7 (C_15_H_18_O_7_), product 8 (C_15_H_22_O_7_), product 9 (C_15_H_20_O_8_), and product 10 (C_15_H_21_NO_9_). Two different structures (called I and II) were devised for product 7. The major individual degradation products of DON in the solid and liquid phases were product 2 and product 7, respectively. The degradation efficacy of CP was much higher in liquid solution than in the solid phase. It was found that ACP treatment reduced the cytotoxicity of both solid and liquid-phase DON in a time-dependent manner. Products 6 (de-epoxy deoxynivalenol) and 3 were coincident with products 2 and 3, respectively, as reported by Chen et al. [48]. Product 4 was reported early [113].

An RMD simulation was performed to theoretically investigate (via computation) the DON detoxification pathways induced by ROS. The simulation results show that O atoms, •OH radicals, H_2_O_2_, and O_3_ can modify toxicologically important functional groups through the ring-opening reaction process of the 12,13-epoxide ring and the scission of the double bond C9=C10, which would reduce DON toxicity [116]. This approach agrees with experimental studies.

LP-DBD plasma treatment using different working gases resulted in T-2 and HT-2 toxin reduction [117,118]. The application of N_2_ as a working gas had a major effect on T-2 and HT-2 toxins. Ten minutes of air-ACP treatment significantly reduced the pure T-2 and HT-2 concentrations by 63.63% and 51.5%, respectively [118]. The mechanism of degradation of T-2 and HT-2 toxins should likely follow comparable pathways to those described or suggested for DON because they have a similar core structure and the same key functional groups (double bond at C9=C10, the epoxide ring, and the OH group at C3). However, further research is needed to identify the degradation products and assess their toxicity in CP-treated foods.

### 6.4. Mechanism of Degradation of Zearalenone

Zearalenone (ZEA) has the formula C_18_H_22_O_5_. It is a macrolide composed of a 14-membered macrocyclic lactone ring fused to a dihydroxybenzene aromatic ring. It can also be degraded by CP, providing less toxic compounds. Zheng et al. [119], after DBD plasma treatment of ZEA and analysis of the degradation products by UHD accurate-mass Q-TOF LC/MS with the ESI conducted in negative mode (ESI-), observed a compound with a molecular formula of C_18_H_22_O_7_ and an *m*/*z* value of 349.1290 [M–H]^−^, which corresponded to a product where the macrocyclic lactone suffers cleavage of the olefinic double bond by O_3_ to form a primary ozonide (1,2,3-trioxolane) following the Criegee reaction mechanism, with the final appearance of two aldehyde groups. The effects of CP-generated ROS on the ZEA molecule may also lead to the product via a different pathway. The degradation followed first-order kinetics and increased with the increase in both treatment time and treatment voltage, in agreement with studies on other mycotoxins.

**Figure 18:**
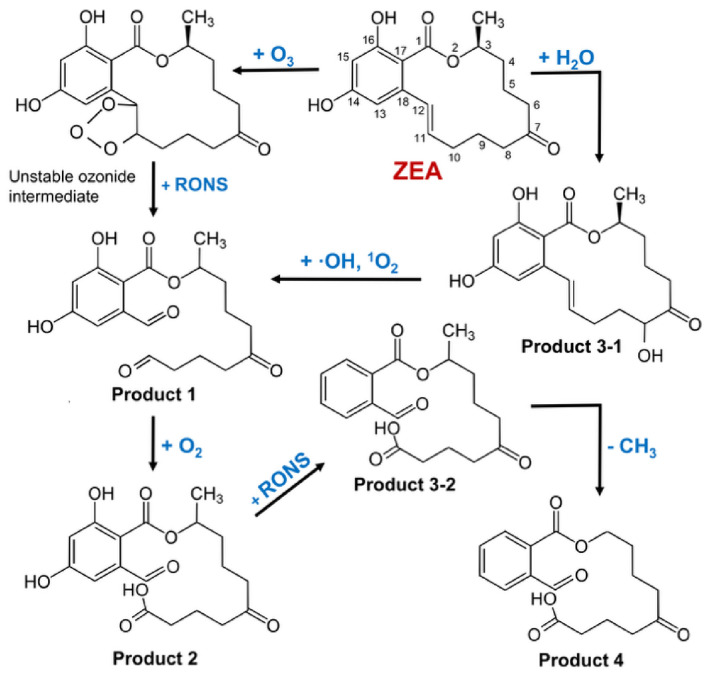
Proposed pathways for the CP degradation of ZEA as suggested by Liu et al. [120].

Liu et al. [120] reported that four major ZEA degradation products were produced after ACP treatment using a dielectric barrier surface micro-discharge (SMD) system due to the oxidative cleavage of the C=C double bond. The degradation rate of ZEA was 96.08% when treated at the maximum power and exposure time (30 W for 3 min). The main degradation compounds identified using LC-ESI(+)-MS/MS were: product 1 (C_18_H_22_O_7_, *m*/*z* = 351.19), product 2 (C_18_H_22_O_8_, *m*/*z* = 367.14), product 3 (C_18_H_22_O_6_, *m*/*z* = 335.14), and product 4 (C_17_H_20_O_6_, *m*/*z* = 321.19), where the *m*/*z* values relate to the protonated [M + H]^+^ ions. The intensity of the degradation products increased from time 0 to 60 s and then decreased with time. Product 1 coincides with the degradation dialdehyde identified by Zheng et al. [119]. The proposed pathway that leads to product 1 through the addition of O_3_ in Figure 18 agrees with that proposed by Zheng et al. [119]. Moreover, product 3 does not have a unique structure because the proposed ZEA degradation pathways assume the formation of a bicyclic product 3-1 and a monocyclic product 3-2 with the same molecular formula, but different structures (Figure 18). Product 2 (C_18_H_22_O_8_), product 3-2, and product 4 have a carboxylic and an aldehyde group instead of the two aldehyde groups observed in product 1. UV light and heat generated by ACP are not energetically sufficient to influence the ZEA degradation. Recently, Zhang et al. [121] highlighted the synergistic effect of ACP and UV light when applied simultaneously on the ZEA molecule. The UV radiation changes the *trans* configuration at the olefin double bond of ZEA to *cis*-ZEA (product 4), while the ACP provides products 1, 2, and 3, with products 1 and 3 being identical to products 1 and 2, respectively, identified by Liu et al. [118]. Product 2 of Zhang et al. [121] is similar to product 1 but has carboxylic groups at positions 11 and 12 of the ZEA molecule.

Wang et al. [122], using RMD simulation, as in the case of other mycotoxins, designed different degradation pathways for ZEA under the activity of ROS produced by CP. They considered the lactone ring, the OH linked to C14, and the C7,8 bond (according to the numbering in Figure 18) as key sensitive groups. However, the degradation products did not match those described by other researchers [119,120].

### 6.5. Mechanism of Action of CP on Patulin

**Figure 19:**
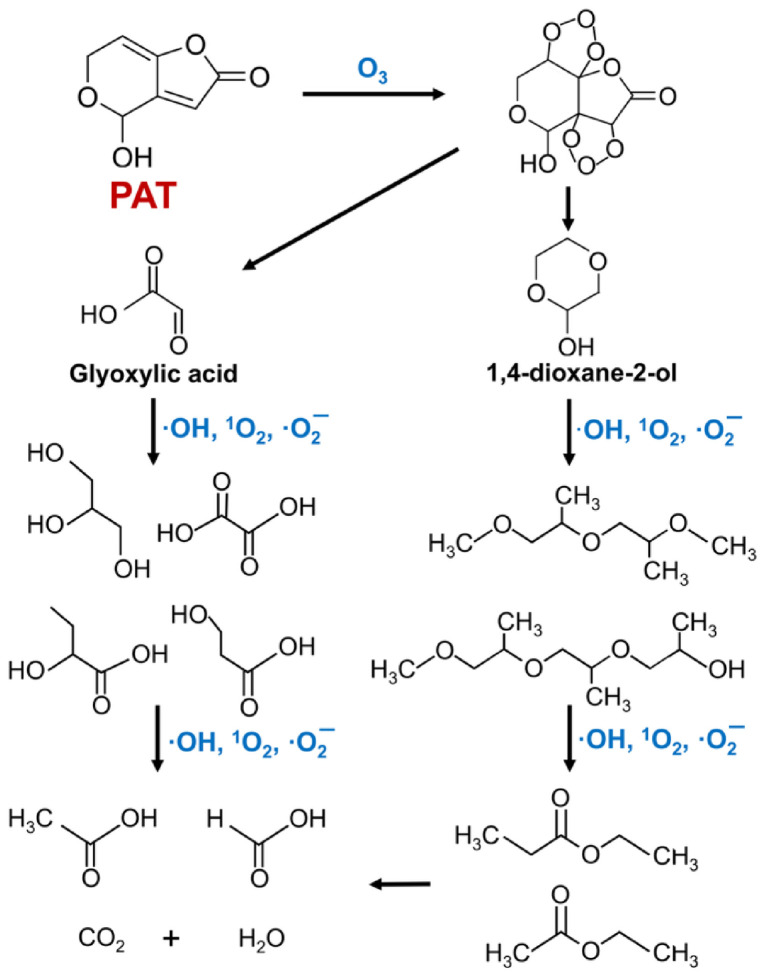
Degradation pathways of PAT under the activity of plasma-activated water (PAW), as suggested by Xue et al. [124].

Patulin (PAT) (C_7_H_6_O_4_) is a bicyclic molecule consisting of a five-membered-lactone ring fused to a six-membered unsaturated hemiacetal ring (4-hydroxy-4H-furo[3, 2-c]pyran-2(6H)-one), widely found in rotten apples contaminated with *P. expansum* but also produced by other species. Production of apple juice from poor-quality apples led to the final product contamination with this toxin [123]. Xue et al. [124] investigated the mechanism of degradation of this toxin under the effect of plasma-activated water (PAW) treatment. The pertinent pathways are illustrated in Figure 19.

The two conjugated double bonds of PAT, one in each cycle, suffer ozonolysis, providing an unstable product that leads to 1,4-dioxan-2-ol and glyoxylic acid after reaction with ROS. The intermediate 1,4-dioxan-2-ol is oxidized to form acyclic byproducts, such as tripropylene glycol monomethyl ether and dipropylenglycol dimethyl ether, which, through further oxidation, would produce ethyl acetate and ethyl propionate. The oxidation of glyoxylic acid leads to some short-chain organic acids and alcohols, which, together with the esters, would be further oxidized to provide acetic acid, formic acid, CO_2_, and H_2_O. The developmental toxicities of intermediate products tripropylene glycol monomethyl ether and glycerol were much lower than those of PAT. These processes were induced by the degradation of PAT on fresh-cut apple slices contaminated with the mycotoxin by DBD CP at 23 kV and are also reported by Shirazi et al. [123]. Moreover, pin-jet CAP fed with air achieved 99% PAT decomposition at 25 kV for 4 min in aqueous solutions [125]. First degradation products of PAT by O_3_ included ascladiol, deoxypatulinic acid, and hydroascladiol as a result of the lactone ring cleavage, which, through further oxidation, should provide 1,4-dioxane-2-ol and glyoxylic acid following the pathway proposed by Xue et al. [124]. The identity of the final low molecular weight products could not be confirmed by LC-QTOF-MS and HR-IM spectra. A complete elucidation of PAT degradation pathways would require further studies involving the identification of multiple transient intermediates. These processes were induced by ROS such as O_3_, some radicals, and hydrated electrons [125].

### 6.6. Mechanisms of CP Degradation of Other Mycotoxins

Although CP systems can reduce the amount of other mycotoxins in foods, few studies have focused on the mechanisms of such degradation [5,90].

Fumonisins are polyketides with characteristic 2-propane-1,2,3-tricarboxylic acids esterified to an aminopolyol chain. CAP can produce the degradation of FB_1_ and FB_2_ in excess of 90% [126]. Large percentages of FB_1_ reduction (64 and 68%) due to CP have been reported in maize spiked with this toxin [55,102]. FB_1_ is highly susceptible to CAP due to its long-chain molecular structure. The FB_1_ molecule consists of a long-chain aminopentol backbone with tricarballylic acid (TCA) side chains attached at the C14 and C15 sites and an amino group attached at the C2 position. Both the amino group and the TCA chains are important toxic groups, which can bind to ceramide synthase and interfere with the synthesis of sphingolipids [120]. The mechanism involves the rapid cleavage of *TCA* side chains and the breakdown of the aminopentol backbone through reactions with RONS [127].

**Figure 20:**
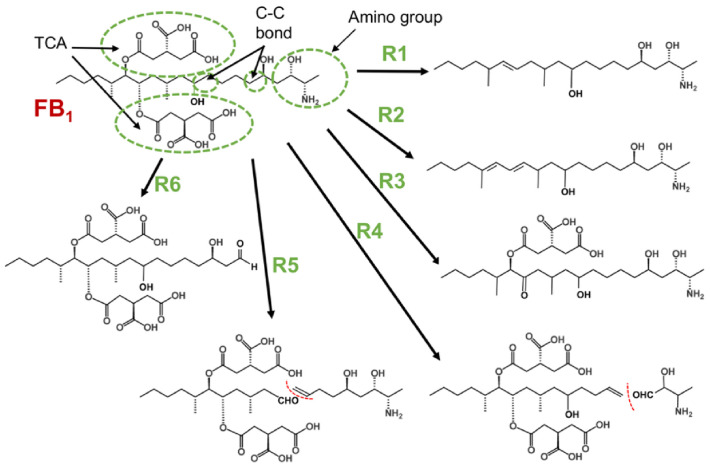
Theoretical degradation pathways of FB_1_ by the effect of CP-generated ROS, as calculated using RMD [122]. The key groups/bonds more likely affected by CP treatment in the FB_1_ molecule are indicated by green dashed lines. The red dashed lines indicate the broken bonds.

Wang et al. [122] used RMD simulation to accomplish a theoretical approach to the possible degradation pathways of six mycotoxins (AFB_1_, DON, ZEA, PAT, OTB, and FB_1_) exposed to the impact of ROS generated by CP. They revealed the probable degradation pathways of these mycotoxins at the atomic level and summarized the reaction types experienced by the main toxic groups, such as addition, oxidation, reduction, ring opening, side chain shedding, and skeleton structure breaking. Some of the intermediates are very reactive and unstable, so they are very unlikely to be identified by conventional high-resolution analytical techniques such as UPLC-QTOF-MS. The possible pathways of FB_1_ degradation by CP are shown in Figure 20. According to the RMD simulation, one or two TCA groups can be removed (reactions R1, R2, and R3), and the C-C bonds adjacent to the OH groups in the aminopentol chain can suffer cleavage with the formation of a double bond and an aldehyde group (reactions R4 and R5). Moreover, the final ethyl amino group can be removed with the formation of an aldehyde group at the C-terminal of the main moiety (reaction R6).

*Alternaria* toxins, such as alternariol and alternariol monomethyl ether, are degraded by the RONS produced by CP [128]. However, the mechanisms of these processes have not been elucidated yet.

## 7. Activity of CP on Mycotoxins in Nuts and Dried Fruits

CP can contribute to mycotoxin mitigation not only by inactivating toxigenic fungi, but also through direct degradation of the mycotoxins already present in food. This aspect is particularly relevant for nuts and dried fruits, where mycotoxins may persist even after fungal inactivation. As summarized in Table 5, CP treatments have shown the ability to modify the molecular structure of these compounds, often leading to less toxic or non-toxic derivatives. However, the extent of degradation varies widely depending on the plasma system, treatment conditions, and, importantly, the presence of the food matrix.

(a) Pistachios

Studies on pistachios (Table 5) highlight both the potential and the limitations of CP for mycotoxin degradation. Reported reductions in AFs range from moderate (about 50%) to high (>80%), depending on the system and conditions applied. For example, air-fed dielectric barrier discharge (DBD) treatments achieved reductions of AFB_1_ of around 50–65% within a few minutes, and higher degradation was observed under in vitro conditions compared to pistachios [83]. Similarly, plasma jet treatments using Ar/air mixtures resulted in considerable reductions, particularly for AFB_1_ (up to 80%), while other AFs (AFB_2_, AFG_1_, AFG_2_) were less affected [6]. This suggests differences in susceptibility among AFs, possibly related to their molecular structure.

A key aspect emerging from these studies is the strong influence of the food matrix. For instance, indirect surface DBD (SDBD) treatments showed relatively low degradation rates in pistachio samples (generally <30%), whereas the same conditions led to much higher reductions in vitro (up to 99% for some AFs) [40]. This disagreement clearly indicates that matrix components can limit the interaction between reactive species and mycotoxins. In addition, processing conditions such as exposure time, distance from the plasma source, and the type of reactive species generated (O_3_ *versus* NOx-dominated regimes) significantly affect the efficacy of the treatment. Greater degradation was observed at shorter distances and longer exposure times, as well as under O_3_-rich conditions. The physical form of the sample also plays a role, as chopped pistachios showed higher reduction rates than whole kernels, likely due to increased surface area and improved accessibility.

CP shows clear potential for reducing mycotoxin levels in pistachios, but the variability in reported results (Table 5) highlights the importance of matrix effects and treatment optimization. These factors remain critical challenges for achieving consistent mycotoxin decontamination.

(b) Hazelnuts

The effect of DBD plasma on AFs in hazelnuts highlights the strong influence of both plasma chemistry and matrix effects (Table 5). In one study [3], treatments using N_2_ and N_2_/O_2_ mixtures showed that pure N_2_ was more effective, achieving >70% total AF reduction after 12 min, with AFB_1_ reduced to approximately 29% at 1150 W. In contrast, the addition of O_2_ decreased efficacy, suggesting that RNS may play a more relevant role than ROS in this matrix. This contrasts with other food systems where O_2_-containing plasmas are often more effective, indicating that optimal gas composition is matrix-dependent. A second study comparing atmospheric pressure (AP) and low-pressure (LP) plasma systems [129] further illustrates inconsistencies across experimental setups. While LP plasma achieved higher reductions in vitro, both systems resulted in similar AFB_1_ reductions (about 70%) in hazelnuts. This discrepancy reinforces the protective role of the food matrix, where lipids and structural components likely hinder the diffusion of reactive species. Moreover, the toxin concentration influenced degradation only in vitro, but not in the hazelnut matrix, again underscoring the limited scalability of in vitro outcomes.

Across studies, AFB_1_ and AFG_1_ were consistently more susceptible than AFB_2_ and AFG_2_, which is typically attributed to structural differences (the C8=C9 double bond present in the former). However, despite similar mechanistic explanations, the extent of reduction varies widely, reflecting the lack of standardization in CP parameters (power, exposure time, gas flow), which makes cross-study comparisons challenging.

(c) Peanuts

As outlined in Table 5, compared to hazelnuts, studies on peanuts show wider variability in outcomes due to differences in reactor design, operating conditions, and sample handling. For example, Devi et al. [4] reported >90% AFB_1_ reduction under a higher energy regime (1950 V/60 W, 12 min), whereas milder conditions achieved only 70% reduction. This confirms that energy input is a key driver of detoxification efficiency, although increasing power raises concerns about product quality and energy cost, which are rarely addressed.

Likewise, reactor configuration plays a critical role. In an APPJ system [130], agitated samples showed higher AF reductions than static ones, indicating that mass transfer limitations are significant in solid food matrices. This is consistent with other studies where improved contact between plasma species and the sample enhances efficacy.

Another study using a rotary plasma jet [87] demonstrated that higher power (200 W versus 180 W) not only improved fast detoxification but also delayed AF reemergence during storage. This introduces an important but often overlooked aspect: post-treatment stability and fungal regrowth, which is rarely systematically evaluated across studies.

Voltage and treatment time were positively correlated with AFB_1_ reduction in a multipin-plane reactor [131], but again, the lack of unified reporting (e.g., energy density, plasma dose) limits comparability. Studies of CP on peanuts emphasize that process parameters and reactor design strongly influence the results, but reproducibility across systems remains unclear.

(d) Roasted coffee beans

In roasted coffee beans, CP achieved only about 50% OTA reduction after 30 min under relatively mild conditions [89], which is notably lower than reductions reported for AFs in nuts (Table 5). This suggests that mycotoxin type significantly affects susceptibility, likely due to differences in chemical structure and reactivity with plasma-generated species. Additionally, the dense and porous structure of roasted coffee beans, combined with their complex chemical composition, may limit the penetration of reactive species. Compared to nuts, this indicates that matrix composition (e.g., porosity, moisture, lipid content) can outweigh plasma intensity in determining efficacy. However, few studies have systematically compared matrices under identical CP conditions, representing a breach in the literature.

(e) Mixed nuts

Studies on mixed nuts (Table 5) provide clear evidence of the matrix effect and differences between contamination types. Dinç et al. [9] showed that AF reductions in spiked samples (AFB_1_, 88.4%) were significantly higher than in naturally contaminated samples (AFB_1_, 24.6%). Likewise, in vitro degradation was even faster, reaching high reduction levels within 2 min. These results demonstrate that plasma efficacy decreases in the order: in vitro > spiked samples > naturally contaminated matrices, due to increasing limitations for the RONS to access mycotoxins. In real food systems, mycotoxins may be embedded within tissues or bound to macromolecules, limiting interaction with reactive species. The study also confirmed that AFB_1_ and AFG_1_ are more reactive than AFB_2_ and AFG_2_, supporting relationships between structure and reactivity. However, despite similar trends, the degradation kinetics varied and required different modeling approaches (Weibull *versus* first-order), suggesting that reaction mechanisms are not uniform across conditions. A crucial limitation of these studies is the lack of standardized contamination models and plasma dosimetry, which complicates comparison and hinders the development of predictive models.

(f) Dried fruits

In dried mulberries, CP achieved up to 62.6% AFB_1_ reduction after 18 min at 13 kV [132], which is moderate compared to reductions reported in nuts (Table 5). This may be due to the high sugar content and sticky surface of dried fruits, which can scavenge reactive species and limit their diffusion. CP studies on dried fruits are scarce, and experimental conditions vary widely, which prevents meaningful comparison. This highlights a significant research gap: the need for systematic studies across different dried fruit matrices under controlled and comparable CP conditions.

## 8. Activity of CP on Mycotoxins in Cereal Grains

Cereal grains are widely contaminated with mycotoxins due to the proliferation of toxigenic fungi in crops, particularly during postharvest stages. Given their central role in global food security (e.g., 1.218 billion tons of maize, 820 million tons of rice, and 798.4 million tons of wheat produced in 2024) [30], effective detoxification strategies are essential. CP has emerged as a promising approach because it can reduce the mycotoxin levels while generally preserving the physicochemical and sensory properties. However, as summarized in Table 6, the reported efficacy varies considerably depending on plasma configuration, mycotoxin type, and grain matrix, highlighting the need for critical comparison.

(a) Barley grains

Studies on barley reveal highly variable and sometimes contradictory outcomes, emphasizing the importance of experimental conditions. Durek et al. [93] showed that the effectiveness of a DCSBD system against OTA strongly depends on the feeding gas: while CO_2_ + O_2_ reduced both fungal growth and OTA levels, CO_2_ alone led to an increase in OTA concentration during storage, likely due to a stress-induced stimulation of OTA production. Similarly, when incubation time before treatment was shortened, OTA levels increased after CP exposure, further supporting the hypothesis that sublethal plasma stress may enhance mycotoxin biosynthesis under certain conditions.

These findings contrast with studies reporting the direct degradation of mycotoxins. For instance, Feizollahi et al. [133] observed up to 48–54% reduction in DON using a DBD system, with only marginal improvements beyond 6 min, suggesting the presence of a reaction plateau. A similar trend was observed for ZEA degradation [134], where efficiency depended strongly on gas composition, with Ar/O_2_ mixtures outperforming air or N_2_. This highlights that reactive species chemistry is a key determinant of degradation efficiency, although the optimal conditions differ between toxins. Further complexity arises from studies using GAD plasma [65], where DON reductions ranged from negligible to 89%, and in some cases, the toxin levels even increased. Notably, the formation of DON-3-glucoside (a masked mycotoxin) was detected, indicating that CP may induce chemical transformation rather than complete detoxification, with potential implications for food safety.

Overall, barley studies demonstrate that CP can both decrease, and under certain conditions, indirectly increase the mycotoxin levels. The high variability and large standard deviations reported in some studies limit the robustness of conclusions, and the short treatment times explored (≤3 min in some cases) further restrict comparability.

(b) Maize grain

In maize, CP efficacy depends on the mycotoxin type and treatment configuration. In vitro studies using DBD plasma [102] showed rapid degradation for some toxins (e.g., enniatin B, FB_1_, OTA, ZEA), whereas DON was much more resistant, with a significantly longer half-life. This indicates that chemical structure governs susceptibility to plasma-induced degradation, a trend also observed in other matrices (Table 6). However, when moving from in vitro systems to maize grains, degradation efficiency decreased substantially. For example, reductions in AFB_1_ and FB_1_ levels were about 65% after 10 min, confirming a matrix-dependent limitation similar to that observed in nuts. This reduction in efficacy is probably due to the limited diffusion of reactive species and interactions with grain components.

Other studies reinforce the importance of process parameters. Increasing the treatment time enhanced AFB_1_ degradation up to 80% after 12 min in contaminated maize [7], while optimization using response surface methodology in a low-pressure N_2_ plasma system [135] predicted reductions above 80% under specific conditions. These findings suggest that optimization of the process can improve outcomes, although such optimized conditions may not be easily scalable.

A comparison of CP configurations further highlights inconsistencies. Somma et al. [55] reported that direct plasma exposure (CP) was generally more effective than plasma-treated gas (PTG), particularly for FB_1_, whereas differences were less pronounced for AFB_1_. Moreover, degradation in maize was consistently lower than in model systems (filter paper), which further confirms the strong influence of the matrix. The identification of multiple degradation products also raises concerns about the toxicity of byproducts, which are rarely assessed systematically.

(c) Wheat grain

In wheat, CP effectiveness varies with both the type of mycotoxin and the treatment conditions (Table 6). For example, *Alternaria* toxins in wheat flour were reduced by 54–74% using an SDBD system [136], with efficiency depending on exposure time and distance, indicating the importance of the geometry of plasma–sample interaction. Similarly, reductions in T-2 and HT-2 toxins up to about 80% were achieved after 10 min of treatment [118], with comparable or slightly lower reductions observed in vitro. This contrasts with other studies where in vitro degradation is higher than in the matrix, suggesting that matrix effects are not always dominant and may depend on toxin localization and sample structure.

For DON, a clear non-linear degradation pattern was observed [79], with a sharp reduction between 3 and 6 min followed by a plateau. This behavior, also reported in other grains, suggests that easily accessible toxin fractions are degraded first, while residual fractions are more resistant.

GAD plasma treatments [47] showed moderate AF reductions (up to 64% for AFB_1_), consistent with trends observed in other cereals. Across studies, AFB_1_ is generally more susceptible than other AFs, but the differences are not always large, again reflecting variability in experimental systems.

(d) Rice grain

CP studies on rice further confirm the strong discrepancy between the in vitro and matrix results (Table 6). Using a corona discharge plasma jet [66], AFB_1_ degradation exceeded 95% on glass slides (in vitro) but ranged from 45% to 57% in rice and wheat, clearly demonstrating restricted plasma penetration and matrix interference. Likewise, Guo et al. [10] reported that DON was more susceptible than OTA, with maximum reductions of approximately 61% and 56%, respectively. While degradation increased with treatment time, overall efficiency remained moderate, suggesting intrinsic resistance of certain mycotoxins and/or protective effects in the grain matrix. Other studies [137] have shown that factors such as moisture content, O_2_ level, voltage, and exposure time significantly influence CP performance. However, even under optimized conditions, reductions in rice rarely exceed 55%, which is lower than in some other cereals. This may be attributed to low permeability and compact grain structure, limiting the diffusion of reactive species.

**Table 6:** Effect of cold plasma on mycotoxins in cereal grains.

Cereal Grain	Type of CP	CP Conditions	Mycotoxin	Effects of CP on Mycotoxins	Ref.
Barley grain	DCSBD 400	Gas: 80% CO_2_ + 20% O_2_, 100% CO_2_, dry air, flow rate: 10 L/min; Power: 350 W; Time: 1 and 3 min	OTA (produced by *P. verrucosum*)	-CO_2_ + O_2_ plasma: The OTA production of *P. verrucosum* was reduced. -CO_2_ plasma: The OTA level was nearly the same directly after the treatment, but was increased after 2 weeks of storage. -Dry air plasma: decreased OTA level when the incubation time of the sample before the treatment was 5 days. However, the OTA level increased when the barley was incubated for only 24 h before the treatment.	[93]
Barley grain	DBD CAP	Gas: air (humid) Voltage: alternating 0–34 kV Power: approx. 300 W Frequency: 3500 Hz; Pulse width: 10 µs; Time: 0, 2, 4, 6, 8, and 10 min; Distance: approx. 2 mm	DON	A significant decrease in DON level within 6 min of treatment was observed (48%). Thereafter, the reduction rate was not significant (54% at 10 min). DON reduction in barley after 6 min ACP increased to 53.6% when the sample was further stored for 24 h, at room temperature.	[133]
Barley grains, canola meal, and canola grain (spiked)	DBD CAP jet	Gas: Ar, Ar/O_2_ (85%/15%), Ar/N_2_ (75%/25%), air, N_2_, N_2_/O_2_ (10%/90%), (20%/80%) Voltage (variable depending on the gas): 0–1 kV, 0–18 kV, 0–22 kV; Frequency: 1 or 3.5 kHz; Time: 1 and 3 min Distance: 2 mm	ZEA, 4 μg/g	ZEA reduction after 3 min in canola grain, canola meal, and barley grains was 91.6%, 83.2%, and 64.8%, respectively. The ZEA reduction rate was influenced by the type of gas mixture and treatment modes (direct or indirect) when the CAP jet was used. Treatment using 85% Ar + 15% O_2_ resulted in the highest ZEA degradation compared to other gas mixtures used. The ZEA reduction rates were not significantly different when air and N_2_ were used.	[134]
Barley grain	Gliding arc discharge (GAD) plasma jet	Gas: air at 5 L/min; Voltage: 3.5 kV; Power: 8.5 W Frequency: 19.2 kHz; Distance nozzle-target: 3 cm Time: 0–30 min	DON	Reduction in DON contamination between 1% and 89%; in 14 samples (70%), the reduction was above 50%. In four samples, an increase between 2% and 33% of DON levels was observed. Likely, DON was converted into D3G after treatment.	[65]
Maize grain and in vitro	DBD jet	Gas: He with small percentages (0.5% and 0.75%, *v*/*v*) of O_2_; Voltage: 6 kV; Frequency: 20 kHz; Distance: 12 mm; Time: 0–60 min (in vitro), 12 min in maize	In maize: AFB_1_ and fumonisin B_1_ (FB_1_). In vitro: AFB_1_, FB_1_, OTA, enniatin B (ENB), ZEA, and DON.	In vitro: All mycotoxins degraded with time, but the rate varied with the type of mycotoxin and the gas composition. Kinetics follows a first-order reaction with a visible reaction plateau for ENB after 20 min. Half-lives range: 1.1–2.6 min for ENB < FB_1_ < OTA < ZEA; 12.4 min for AFB_1_; ≈74 min for DON. In maize: After 10 min, AFB_1_ and FB_1_ were reduced by 65% and 64%, respectively.	[102]
Maize grain	DBD	Gas: ambient air, RH 50–55%; Voltage: 120–160 kV; Frequency: 50–70 Hz; Distance: 30 mm; Time: 2, 4, 8, and 12 min	AFB_1_ (produced by *A. flavus*)	After treatment for 2, 4, 8, and 12 min, the AFB_1_ content in maize was significantly reduced; it decreased by 23.44%, 41.80%, 64.44%, and 79.98%, respectively.	[7]
Maize grains (spiked) and in vitro on filter paper	DBD: close plasma (CP), PTW, and PTG	Gas: pure O_2_ and synthetic air; Voltage: 13 kV; Frequency: 6 kHz; Distance: 2 mm; Time: 3 and 20 min	AFB_1_ and FB_1_	AFB_1_: Time was relevant for degradation. The maximum degradation (23% on maize kernels; 77% on paper) was achieved after 20 min using PTG-O_2_. FB_1_: Time was not irrelevant for degradation. A 20-min CP-O_2_ caused the highest degradation in maize grains (68%), while in paper, the highest degradation (78%) occurred at 3 min.	[55]
Maize	Low-temperature nitrogen plasma (LTNP)	Gas. N_2_ at low pressure 0.1–1.7 Pa; Power: 60–200 W; Time: 5–1800 s	Total AF at initial concentrations of 66.12–105.98 ppb	A response surface methodology model found that an increase in exposure time led to a significant decrease in AF concentration. To achieve a reduction in AF content of 82.6%, a time of 1793.4 s, a pressure of 0.98 Pa, and an ionization power of 189.8 W is required.	[135]
Wheat flour (spiked)	SDBD	Gas: air, with RH = 45% Voltage: 2340 V; Frequency: 50 Hz; Times: 30, 60, 90, 120, 150, and 180 s; Distance: 6, 21, 36, and 51 mm.	*Alternaria* toxins: alternariol (AOH), alternariol monomethyl ether (AME), and tentoxin (TEN)	The maximum reduction in *Alternaria* toxins was obtained by treatment performed at the minimal distance (6 mm), and at the highest exposure time (180 s), resulting in reductions of 60.6%, 73.8%, and 54.5% for AOH, AME, and TEN, respectively.	[136]
Wheat grain (spiked) and in vitro	CAP	Gas: air; Voltage: 80 kV RMS; Frequency: 50 Hz; Times: 0, 5, and 10 min; Distance: 4 mm	T-2 and HT-2 toxins	In wheat grain, 10 min of air-CAP treatment significantly decreased the T-2 and HT-2 concentrations by up to 79.8% and 70.4%, respectively. In vitro, air-CAP reduced the T-2 toxin concentration by 63.63%, and HT-2 toxin by 51.5%.	[118]
Wheat grains and in vitro	Dielectric barrier surface micro-discharge (SMD)	Gas: air; flow-rate 1 L/min; Voltage: 4 kV; Power: 5 ± 0.15 W; Frequency: 7 kHz; Distance: 3 mm; Time: 0–3.5 min (in vitro); 3, 6, 9 min (wheat grains)	DON produced by *F. graminearum*	In vitro CP can reduce DON production. DON contents decreased with treatment time, but there were no significant differences among the 1, 2, and 3-min treatments. In wheat flour, DON contents decreased by 6.4%, 52.9%, and 54.7% after 3, 6, and 9 min, respectively. In dry mycelia of *F. graminearum*, DON contents decreased by 30.0% 48.4% and 48.5% after 3, 6, and 9 min treatments, respectively.	[79]
Wheat grains (and in vitro)	Double dielectric barrier discharge (DDBD) cold plasma	Gas: air at ambient pressure; Voltage: 40–160 V; Frequency: 140–220 Hz; Duty cycle: 20–100%; Time: 15 min (wheat) and 35 min (in vitro)	DON (initial concentration 0.5 to 5 µg/mL)	DON degradation in solution reached 98.94% after 25 min under optimal conditions. About 61% DON reduction in wheat after 15 min plasma treatment.	[114]
Wheat grains	GAD CP	Gas: dry air (21% O_2_, 79% N_2_) (2.5 L/min) + moist air (130 mL/min); Voltage: 2, 2.5, 3, and 3.5 kV; Power: 5.39, 6.88, 8.98, and 10.88 W; Frequency: 20 kHz; Time: 2, 6, and 12 min	AFs	In general, the treatment of 10.88 W power and 12 min of treatment showed the highest performance in the reduction in the levels of AFB_1_ (64%), AFB_2_ (41%), AFG_1_ (59%), AFG_2_ (40%), and total AF (61%).	[47]
Wheat (spiked with DON) and in vitro	Electron cyclotron resonance microwave (MW-ECR) plasma (2.45 GHz) plus radio-frequency capacitively coupled plasma (RF-CCP) (13.56 MHz)	Gas: air, flow-rate: 50 mL/min (normal conditions): Pressure inside the chamber: 1 Pa; Wheat layer height: 3 mm, placed on a grid approx. at the chamber center	DON (initial concentration: 1161 ppb)	After 60 min, half of the DON was degraded, resulting in a concentration of 622 μg/kg. 98% degradation observed on the glass substrate compared to 50% on the wheat sample	[138]
Rice, wheat grains (spiked), and in vitro	CDPJ	Gas: air Voltage: 20 kV DC Frequency: 58 kHz; Distance: 15, 25, and 35 mm Time: 0–30 min	AFB_1_	On glass slides, the initial concentrations of AFB_1_ were decreased by >95% in 30 min. In spiked cereal grains, AFB1 levels in rice and wheat were reduced by 56.6% (rice) and 45.7% (wheat) in 30 min.	[66]
Rice grain (artificially contaminated with mycotoxins)	DBD	Gas: not specified. Voltage: 25 kV; Distance: 20 mm Time: 0, 2, 4, 6, and 8 min	DON and OTA	The reduction % increased with treatment time, and at each time, it was higher for DON than for OTA. The maximal reduction in DON and OTA was 61.25% and 55.64%, respectively, at 8 min.	[10]
Rice inoculated with *A. parasiticus*	DBD	Gas: supposed air; Voltage: 28–169 kV, Frequency: 50–200 Hz; Time: 5–60 min	AFB_1_ and total AFs. Initial levels: 66.12 μg/kg and 208.58 μg/kg, respectively	After being treated for 60 min, the degradation rates of AFB_1_ and total AFs were 55.34% and 52.61%, respectively. The degradation rates increased with the applied voltage. The AFs were naturally produced by *A. parasiticus*.	[137]

Generally, CP shows promising potential for mycotoxin mitigation in cereal grains, but its efficacy is highly variable and difficult to compare across studies. The existing evidence shows that matrix effects are a major limiting factor, as degradation is consistently lower in real grains than in in vitro systems due to the restricted diffusion of reactive species and interactions with the grain components. In addition, mycotoxin structure influences susceptibility, with DON generally being more resistant than AFs and related compounds. Although increasing treatment time, power, or modifying gas composition can enhance degradation, the effects are often non-linear and approach plateaus, suggesting limited accessibility of residual toxins. Notably, adverse outcomes have been reported, including increased mycotoxin levels due to fungal stress responses and the formation of masked mycotoxins, which may avoid complete detoxification. A key limitation of the current literature is the lack of standardized CP parameters and reporting metrics, which hampers reproducibility and scalability. Future work should prioritize standardized methodologies, a comprehensive evaluation of degradation products, and their toxicity.

## 9. Effect of CP on the Physicochemical and Organoleptic Properties of Nuts, Dried Fruits, and Cereal Grains

It is very important to verify the impact of treatments on the physicochemical quality of the foods after being exposed to CP. According to most reports, there are minimal negative influences on their physical features. However, depending on the values of some of the key parameters of the CP systems, changes in the sensorial characteristics may be observed.

### 9.1. Effect on Nuts and Dried Fruits

After AP-CCP exposure of pistachios, at a stage with 100 W power input, a sensory panelist team did not observe differences in terms of the overall appearance, color, odor, and texture. However, at a stage with 150 W, the panelists observed slight alteration only in the texture of treated pistachios [81]. However, Makari et al. [83] observed that while the total phenolic content (TPC) of pistachios was not affected by the DBD plasma, the antioxidant activity was slightly stimulated, although there was no significant difference between treated samples at exposure times longer than 60 s. The malondialdehyde (MDA) contents increased with the increase in the treatment duration and displayed the highest increment after 180 s. The levels of chlorophyll A and B and total carotenoids decreased. Color parameters were affected, and plasma treatment led to darker pistachio nuts. Protein solubility was reduced, and the pattern and intensity of proteins in the pistachio nuts were altered. Plasma treatment induced positive and negative effects on the quality features of pistachio nuts, but quality changes were lower than those of other decontamination technologies. Esmaeili et al. [6] reported that a panel of experts found that the plasma jet had little effect on the color, taste, and smell of treated pistachios. The peroxide value increased very slightly (from 0.07 to 0.08 meq/kg), and the moisture content decreased slightly, concluding that changes induced by CP were minimal. Hydroperoxides, thiobarbituric acid reactive substances, phytosterol oxidation products, and C6–C10 aldehydes and alcohols, key markers of lipid peroxidation, were observed in SBDB-treated pistachio kernels. However, their amount was not significantly different between the treated and untreated samples and caused insignificant changes in the total composition of kernel lipids (fatty acids, or alcoholic constituents of unsaponifiable matter) [139].

The results of a sensory evaluation of hazelnuts treated by AP CP by a panel of experts did not show significant differences between the CP-treated and untreated control samples [84]. AP plasma-treated hazelnuts were found to be organoleptically acceptable.

An increase in the TPC and antioxidant activity after 15 and 30 days of storage at 4 °C was observed in fresh and dried walnut samples used in both the control and samples treated with plasma jet fed with Ar at a distance of 1.5 cm. Changes in the total phenolic content and antioxidant activity in the control sample and treated walnuts showed a similar pattern [86]. No change in the peroxide value but a slight decrease in the moisture content of dried walnut kernels from 3.38% to 2.42%, together with a darkening in color after LP-CP treatment at 50 W for 20 min, was reported in another study [140].

Devi et al. [4] indicated that after the rotary plasma jet exposure of peanuts, there was no generation of free fatty acids (FFAs), even after using 200 W for 5 min in the peanut oil produced from them. In terms of peroxide value, after 180 W or 200 W treatment for 3.5 min, the value increased slightly, but there was no significant difference from the control group. However, after 180 W and 200 W treatment for 5 min, the peroxide value (PV) increased from 3.04 meq/kg to 5.36 and 5.62 meq/kg, respectively, which resulted in significant increases and was explained by the increase in the surface temperature of the peanuts when the time increased from 3.5 to 5 min. ROS from plasma may act at relatively low temperatures, but rise with time, on the double bonds of the fatty acid chains and oxidize them, forming peroxides that cause rancidity of the derived peanut oil. No changes were observed in the hardness and fracture or shearing force of CP-treated peanuts compared to the control samples [88], and only a slight variation in the color of the peanut oil was observed. Only non-significant changes in the color parameters or the PV have been reported in peanuts [87,88]. Iqdiam et al. [130], using an APPJ system, found no significant differences in the acidity and PV of oil produced from their peanuts treated with short times (1–2 min) without shaking. Treatments on agitated samples and times of 3–5 min produced a lower surface temperature, which does not adversely affect the oil quality. Several attributes, including overall appearance and color, were considered by panelists tasting the peanut samples. There were no significant differences in overall appearance preference for the peanut samples, and CP exposure may actually enhance the sensory quality of the peanuts. A reduction in the moisture content of peanuts following CP treatment at 30 kV for 15 min has been reported [131]. The reduction in moisture content may be due to the breakdown of water molecules into oxygen-free radicals during the CP treatment process. The fat, protein, and fiber contents increased with longer durations at higher voltages of CP treatment. The highest fat content was observed after 15 min at 30 kV. On the contrary, the carbohydrate content gradually decreased as the exposure time and voltage of CP treatment increased due to starch depolymerization. The mineral content (Ca, Fe, Zn) showed slight changes depending on the treatment. The total content of phenols and flavonoids increased with CP treatment, although it decreased with prolonged exposure to high voltages. The effect of APCP on the physicochemical features of mixed nuts (roasted white chickpeas (23.4%), roasted yellow chickpeas (10.2%), pistachios (12.9%), hazelnuts (16.3%), pumpkin seeds (16.2%), and peanuts (21%) was studied [9]. They found that after CP exposure, moisture decreased, while the FFA and peroxide values were slightly augmented, and the intensity of the changes depended on the time; other quality parameters (protein, fiber, ash, and mineral content) remained stable.

CP showed a minimal impact on the color of dried white mulberries but caused decreases in pH (from 5.18 to 4.12) and TPC (by approximately 32%), indicating some quality degradation [132]. Wang et al. [109] treated raisins with surface discharge plasma CP. The key attributes, such as moisture content, titratable acidity, total sugars, ascorbic acid content, color parameters, and aroma in raisins, showed no significant changes after plasma treatment.

### 9.2. Effects in Cereal Grains

CP modified the physicochemical and sensory properties of cereal grains. This treatment affects the structure of starch and proteins as well as their appearance.

The color suffers some changes depending on the treatment conditions. Using the CIE L*a*b* color system parameters, it has been reported that in maize, L* and b* decreased slightly while a* increased under glow discharge plasma using Ar and N_2_ as feeding gases at the time the antioxidant capacity (total phenol, ascorbic acid, and glutathione) was enhanced, while the FFA value and MDA content diminished during storage for 180 days [72]. Little changes in the activity of DBD on maize grains were reported by Zheng et al. [141]. No significant change in crude protein content was detected; fatty acids increased a little after treatment, while the crude fiber content decreased slightly. In wheat grains, no significant impact on physiological properties, except for minor changes in the moisture content, a* value, and MDA levels, was observed [110]. Negligible effects on wheat quality were observed after DBD CP treatment, except for a slight decrease in the whiteness of wheat powder. There were no significant differences compared to the controls in vitamin B1 and vitamin E concentrations, moisture content, and soluble protein content [48]. CP-generated RONS can interact with starch, promoting polymerization or depolymerization, plasma etching, and attaching nitro, hydroxyl, carboxyl, and other groups, which alter features of starch such as viscosity, crystallinity, and gelatinization temperature [142,143]. Color analysis of wheat flour treated with CP revealed a slight increase in lightness and whiteness index attributed to ROS oxidative effects on pigments. Moreover, significant enrichments in swelling power, solubility, damaged starch, and water and oil absorption capacities with increased plasma exposure time pointed to structural modifications in the starch and protein contents [144]. CP etching of maize starch can increase the particle size, disturb crystalline structures, and break molecular chains, which provide lower viscosity and improve flow characteristics [143]. Concerning the proteins of cereals, CP-generated ROS can oxidize free amino acids and protein-bound residues. These changes include the hydroxylation of side chains, nitration of aromatic groups, S-nitrosylation of thiol groups, sulfoxidation of methionine, chlorination of amino groups, and the conversion of amino acid residues into carbonyl derivatives. Therefore, the secondary and tertiary structure of proteins and their functionalities can be modified [143]. In barley grains, albumin, globulin, and prolamin were hydrolyzed, producing smaller peptides or free amino acids [145]. Lipid peroxidation was observed in wheat exposed to DBD plasma [110]. As indicated by Zhen et al. [141], the levels of some fatty acids were increased in maize after DBD CP treatment, especially at 50 KV for 120 s, though the changes were considered within acceptable limits for storage, and no significant change in the crude protein levels was observed. Thus, the effect of CP treatment on maize quality is mostly adequate, and it can even improve the quality in some aspects, and CP has an application potential in the processing and storage of maize [141]. Iqdiam et al. [118] indicated that their air-CAP treatment of wheat grains did not significantly alter the wheat grain quality, color, or germination, suggesting its potential for food safety applications. A DBD CP had no significant impact on rice quality except for fat oxidation, and the FFA value of rice increased by 275.3% under a voltage of 160 kV [137]. A DBD CP treatment of rice grain (2–8 min duration) did not significantly influence the rice grain moisture content of the sample within an exposure duration of 8 min [10]. CP treatment only exerted a significant influence on the cell membrane permeability of the rice grain when a certain treatment duration (8 min) was reached. The FFA content was increased due to the hydrolysis of triacylglycerol and phospholipids. Oxidation of the double bonds of FFA under the activity of lipoxygenases increases the amount of lipid peroxides that, by cleavage, provide aldehydes and ketones, and an increase in the MDA content. However, the results showed that the CP treatment did not influence the major nutrients of rice grain. Moreover, the DBD CP treatment for 8 min or less did not influence starch and amylose content in rice grain. CP had no significant influence on the globulin, glutelin, or albumin content of rice grain but significantly affected the prolamine content. However, overall, CP treatment has a negligible effect on rice protein. Fundamental CP parameters such as the nature of the feeding gas, power, and exposure time are critical in balancing oxidative effects with the preservation of quality in cereal grains. CP has the potential to modify the structure of food allergens, which may reduce their allergenicity, though this is still a developing area of study [143]. It may be concluded that compared to other methodologies, the CP treatment of these food commodities does not change their organoleptic and physicochemical properties much, provided that the CP conditions are not severe and the exposure time is relatively short.

### 9.3. Implications of the Matrix of Low-Moisture Foods in the Industrial Scalability

Considering the variable composition and structure of the food objects of this review, the possible scalability to the food industry has to deal with several aspects of CP. One of them can be the matrix effect of these products, summarized in their low moisture, rough surface topography, and high fat or protein content. Low water activity inside the nuts and grains makes the production of RONS difficult compared to fresh foods. Rough surfaces in nuts and seeds imply that crevices, porosity, and irregularities can harbor microorganisms that are more challenging to reach by the reactive species produced by CP, which usually implies larger treatment times. An adverse effect of CP relies on the surface etching of the nuts and seed shells, which can increase the permeability of microorganisms to reactive species but may also increase the surface roughness or enhance wettability. Grains and especially nuts are rich in unsaturated fatty acids. While the CP nature preserves most nutrients, the ROS can activate lipid oxidation, leading to increased peroxide values and potential rancidity, particularly in peanuts and pistachios, as previously commented. In the case of dried fruits, the high concentration of sugars creates a dense, viscous, and often sticky surface matrix, which physically traps microorganisms or spores, making them significantly more resistant to plasma active species compared to dry surfaces. CP can also modify protein structures in these foods, as previously discussed, potentially mitigating allergenicity by breaking down or altering the protein structure through oxidation in peanuts (variety Ara h1) [146] or depolymerization of the proteins gliadin and glutenin in wheat flour (glutenin) [147].

The matrix effects generate chances and challenges for industrial implementation. Thus, as previously indicated, CP is highly efficient at the surface decontamination of dried nuts and grains, achieving 1–5 log reductions of *Aspergillus* sp. with minimal impact on nutritional and organoleptic quality. As the surface is the target, CP is ideal for in-package decontamination, preventing re-contamination during packaging. CP can increase the functionality of grain flours by altering starch granules and increasing the water-holding capacity, which is desirable in baking. It also helps in decreasing the cooking time of grains. The type of CP equipment (DBD, APPJ, GAD plasma, MW plasma), the exposure time, power/voltage input, the frequency, gas mixtures (e.g., Ar, He, N_2,_ or air), and gas-flow rate must be appropriate to the specific food to be processed to avoid undesirable changes, such as the darkening of nuts or flavor alterations (e.g., rancidity, off-flavors) due to oxidation. This process needs an optimal balance of power and treatment time.

While effective at the laboratory scale, the industrial implementation of CP in low-moisture commodities such as nuts, cereal grains, and dried fruits still presents important technical and economic challenges. Large-scale applications require robust and cost-effective plasma systems with optimized reactor designs capable of uniformly treating bulk products under continuous processing conditions. In dielectric barrier discharge (DBD) systems, operating parameters such as applied voltage, gas composition, electrode configuration, barrier material, geometry, and electrode wear must be carefully optimized according to the characteristics of each food matrix [148,149]. Treatment uniformity is particularly challenging in bulk dry products because heterogeneous particle distribution and limited surface exposure may reduce the contact between RONS and contaminated sites. Moreover, as previously indicated, the matrix composition strongly influences CP efficiency.

Although CP has shown promising results for mycotoxin mitigation in laboratory studies, its industrial translation remains limited. Major constraints include the scale-up of plasma generation systems, maintenance of homogeneous treatment in large and heterogeneous dry food matrices, integration into existing industrial processing lines, and the high initial investment associated with industrial plasma equipment. In addition, process standardization, energy efficiency, economic feasibility, and regulatory approval of CP-treated products must be addressed before widespread commercial adoption. Therefore, further pilot-scale investigations focused on low-moisture food systems are required to optimize processing conditions and facilitate the industrial implementation of CP technology.

## 10. Toxicological Considerations of Cold Plasma-Treated Foods: Mycotoxin Degradation and Food Matrix Effects

CP treatment raises important toxicological considerations that extend beyond the degradation of mycotoxins to include potential modifications of intrinsic food components. Accordingly, the safety of CP-treated foods must be evaluated from two complementary perspectives: (i) the nature and toxicity of mycotoxin degradation products, and (ii) the formation of reactive or altered compounds within the food matrix, such as oxidized lipids, modified proteins, or carbohydrate-derived products, as previously indicated in Section 9.

The toxicological implications associated with the degradation of major mycotoxins are discussed below.

AFB_1_ can be metabolized and activated by CYP450 enzymes in the liver. This process leads to the formation of several toxic metabolites, such as AFB_1_-8,9-endo-epoxide or AFB_1_-8,9-exo-epoxide. These intermediate compounds are highly toxic and can be detoxified by enzymatic conjugation with reduced glutathione, giving AFB_1_-glutathione, a water-soluble derivative that can be excreted in bile and urine. Moreover, they can be partially hydrolyzed to AFB_1_-dihydrodiol, which is quite polar and can be excreted or can bind lysine residues, leading to protein damage and necrosis, thus representing an additional toxic mechanism of AFB_1_ [150]. In the case that they are not eliminated, they can bind to intracellular proteins, DNA, and RNA, producing gene mutations resulting in cytotoxicity and carcinogenesis [151,152]. Specifically, they can bind the N-7 position of guanine residues in the DNA molecule, which produces a highly unstable adduct that may induce hepatocellular carcinoma [153,154]. The furofuran ring, and particularly, the double C8=C9 bond on the terminal furan ring of AFB_1_, are considered primarily responsible for its toxicity and carcinogenicity. Any changes in the furofuran ring, the lactone ring, the cyclopentenone, or the methoxyl structure at C4 would result in a marked reduction in the biological activity of AFB_1_ [104]. Plasma-generated ROS can react with key groups of the AFB_1_ molecule and thus contribute to a decrease in its toxicological effects [55]. Thus, exposure of aflatoxin-containing foods to CP produces many products where the molecular groups or bonds responsible for the harmful effects of these mycotoxins disappear, providing many other products that are likely to be less toxic than AFs. Human hepatoma carcinoma cells (HepG2) constitute an appropriate in vitro model for aflatoxin toxicity assays. The formation of DNA strand breaks in HepG2 cells as a result of the genotoxic activity of CAP-exposed AFB_1_ samples has been assessed [105,154]. CP-treated AFB1 showed a decreasing trend in the formation of DNA strand breaks with increasing treatment time, regardless of the RONS dose used [105]. The products from the degradation of CP-treated AFB_1_ showed lower or null toxicity compared with untreated AFB_1_ in HepG2 cells [7,102,105,154]. The removal of the key double bond in the AFB1 terminal furan ring by CP-generated RONS contributes to the reduced mutagenicity of AFB_1_; however, the opened dialdehyde ions may form Schiff bases with primary amine groups in proteins, occasioning protein adducts responsible for the additional cytotoxic effect of AFB1 [153]. CP modification of the AFB1 active site induced the production of AFB2a and AFB1-diol, which are cytotoxic but can be degraded, similarly to AFB1.

Concerning DON, its key functional groups related to toxicity are the unsaturated bond at C9=C10, the 12,13-epoxy ring, and the hydroxyl group at C3. The CP-produced RONS, like O_3_, can modify or eliminate these groups, and, therefore, the produced compounds are likely less toxic than DON [111,112,113,155]. The reduction in the cytotoxicity of AFB_1_, DON, and NIV in mouse RAW264.7 macrophages after treatment by MW plasma at atmospheric pressure has been reported [156]. Ott et al. [157] also reported a decrease of 80% in the cytotoxicity in colon carcinoma Caco-2 cells of HV CAP-treated DON in liquid solution after exposure at 85 kV for 20 min, although the cytotoxic reduction in solid DON was not significant, probably due to the increased availability of reactive species provided by water, such as O_3_, H_2_O_2_, and others. Further tests using a human-derived renal proximal tubular cell line (HKC-8) found that the cytotoxicity of CAP-treated DON was significantly reduced compared with that exhibited by the untreated controls [115]. The toxicity of the degradation products of CAP-treated ZEA in cereals was assayed both in vitro, using human normal liver cells (L02), and in vivo, using male C57BL/6J mice [120]. It was found that the toxicity of ZEA was significantly reduced by the treatment, which indicates that the degradation products are practically non-toxic or slightly toxic. Low toxicity of OTA-containing roasted coffee beans after exposure to CP has been reported [89] using *Artemia salina* (brine shrimp) as the animal test for in vivo assays. Zheng et al. [110] corroborated this decrease in the toxicity of CP-treated OTA in vitro using HepG2 cells. When these cells were exposed to CP-treated OTA, their viability increased significantly compared with exposure to the control non-exposed OTA (from about 50.8% to about 94.7% viability under optimized conditions). This suggests that the cold plasma degradation products of OTA are much less toxic to HepG2 cells than untreated OTA, indicating effective detoxification in a relevant human liver cell model. Thus, in vitro and in vivo experiments have revealed that the toxicity of CP-treated mycotoxins is effectively reduced. The lower toxicity of the degradation products of AFB_1_, DON, or ZEA after CP exposure is likely due to the changes undergone in the key groups linked to their toxicological effects [102,104,111,112,113,114,158]. It is critical to recognize that mycotoxin degradation does not automatically guarantee complete detoxification, because some of the primary degradation products may undergo further reactions, such as nitration or oxidation, which can increase their toxicity. Thus, a systematic assessment of the degradation pathways and the toxicity of the subsequent products is essential [102].

More comprehensive research must be conducted on CP-induced physicochemical changes of food products and their subsequent impacts on in vitro/in vivo toxicity. Overall, current research has generated different results concerning the cytotoxicity and genotoxicity of CP-treated products. Nastasa et al. [159] evaluated long-term contact with PAW in CD1 mice. The mice received PAW containing NO_3_^−^ (1550 mg/L) and H_2_O_2_ (2.6 mg/L) or tap water daily for 90 days as the sole water source. The results indicated no adverse effects on mice during the experimental period. In agreement with these results, Xu et al. [160] evaluated the safety of plasma-activated liquids (PALs) in New Zealand rabbits administered by intra-bone marrow injection, where acute toxicity tests showed that there were no PAL-related side effects in rabbits receiving a maximum dose of 700 μL PAL containing up to 500 μM H_2_O_2_ (17 mg/L) concentration; thus, no mortality or loss of mobility was reported, which provided a basis for the further clinical research and application of PAL.

Some in vitro studies indicate that there is no significant alteration in the toxicity in human keratinocytes [161,162]. However, low cytotoxic effects were detected in vitro using the Chinese hamster ovary cell line CHO-K1, although mutagenic events were likely spontaneous mutations [163,164]. Other researchers have found increases in CHO-K1 toxicity [165] or in human fibroblast cells (GM00637) [166]. In vivo assays also show different behavior depending on the tested species and the conditions used for the experiments. Hesling et al. [163] reported that *Galleria mellonella* larvae injected with lettuce broth exposed to CP were negatively affected depending on the time conditions, which was related to the polyphenol compounds of the lettuce broth that can influence the results. Nevertheless, no significant toxicity increase was reported in the red flour beetle *Tribolium castaneum* [165], rats [164], Sprague–Dawley rats [167], or mice [159,168]. PAW delayed the recovery time or even resulted in a lack of growth of *Danio rerio* (zebrafish) embryos compared to control samples if the RONS concentrations were higher than the defined threshold values [169]. Plasma-activated (at 1 kHz) cashew apple juice caused significant lethality and morphological abnormalities at a concentration of 1000 μg/mL on *Artemia salina* nauplii, while no toxicity was observed at 10–100 μg/mL, indicating dose-dependent toxic effects [170]. Nevertheless, the same group of researchers further reported no toxic effects of the same juice treated by DBD plasma at lower frequency (400 and 550 Hz) in *A. salina* instar II nauplii and zebrafish for both embryos and larvae (for 24 and 48 h), showing that this juice is toxicologically safe for human consumption, once CP processing has not produced substances at concentrations that could be harmful [171]. Male and female Sprague–Dawley rats administered orally with CP-treated edible films for food packaging at doses of 5000 or 1000 mg/kg body weight did not show signs of acute toxicity or death after 14 days of observation. Although changes in the levels of several hematological components of samples were observed, the changes compared to the control were considered to be toxicologically irrelevant as their levels were within normal physiological ranges [167]. The primary source of toxicity is the interaction of RONS with biomolecules (proteins, carbohydrates, or lipids), which can alter their structure and functionality [163].

These disagreements in toxicological assessments are essentially due to variations in CP systems and treatment parameters. There are critical factors affecting the safety of CP-treated food: exposure times >5 min can increase RONS concentration, which may exceed the cell tolerance levels. Solid foods, such as nuts or grains, usually show less marked chemical modifications than liquid matrices because the treatment is mainly limited to the surface. In dried fruits rich in sugars, a large exposure time can increase the level of the toxic degradation byproduct hydroxymethylfurfural. The power and voltage influence the concentration of RONS generated. High values increase the intensity of the treatment. The generation of compounds like H_2_O_2_ or nitrite is transitory and generally dissipates quickly or remains well within safe levels. Concerning the low-moisture commodities object of the present review, there is a large gap in studies on the toxicology of these foods when administered in vivo or in vitro after being treated with CP. One of these scarce studies is that of Los et al. [165]. The residual toxicity of ACP treatment of grains for food purposes was assessed by feeding beetles (*Tribolium castaneum*) with flour produced from DBD plasma-treated wheat grains [165]. No negative impacts on the survivability or weight profiles of insects were observed. Assessing the potential toxicity of CP-treated foods is crucial to ensuring the safe development of processes and commercial applications in the food industry. Establishing the safety of mycotoxin degradation products is of paramount relevance for the regulatory approval and consumer acceptance of CP technology.

Despite the generally observed reduction in cytotoxicity and genotoxicity following CP treatment, the toxicological safety of degradation products cannot yet be considered fully established. Most available studies rely on in vitro assays or short-term in vivo models, which may not adequately capture long-term or cumulative effects. In addition, the chemical structure of many intermediate degradation products remains insufficiently characterized, and their toxicity is often inferred rather than directly assessed. This variability further limits the establishment of general conclusions.

Therefore, systematic identification of degradation pathways, coupled with comprehensive toxicological evaluation, including long-term in vivo studies, is required to ensure the safety of CP-treated foods before large-scale application. Particular attention should also be given to CP-induced modifications of intrinsic food components (e.g., lipid oxidation products or structurally altered proteins), even in the absence of mycotoxins.

## 11. Conclusions and Future Perspectives

Cold plasma (CP) has emerged as a promising non-thermal technology for improving the microbiological safety of low-moisture foods, including nuts, dried fruits, cereal grains, and flours. The studies reviewed here demonstrate that CP can effectively inactivate toxigenic fungi and reduce the concentration of major mycotoxins, including AFs, OTA, DON, ZEA, patulin, and *Alternaria* toxins. These effects are mainly attributed to the action of RONS generated during plasma discharge, which contribute to both fungal inactivation and to the chemical degradation or transformation of mycotoxin molecules.

A major key point from the current literature is that CP efficacy is highly matrix-dependent. Food composition, surface characteristics, moisture content, and lipid levels strongly influence treatment performance and product stability. While CP treatments can achieve substantial microbial and mycotoxin reductions, excessive exposure may promote lipid oxidation, rancidity, off-flavor development, or modifications of proteins and carbohydrates. Thus, the optimization of treatment parameters for each specific product is needed to balance decontamination efficiency with the preservation of nutritional and sensory quality.

Another important conclusion is that the lack of method standardization remains a major limitation for the field. Large variability among CP systems, reactor configurations, feeding gases, voltage and power inputs, and other treatment conditions makes a direct comparison between studies difficult and complicates the establishment of generally applicable protocols. In addition, most studies have been conducted under laboratory conditions using artificially inoculated samples, which highlights the need for validation under industrial conditions and naturally contaminated matrices.

The available studies also indicate that CP-induced mycotoxin degradation does not necessarily guarantee toxicological safety. Although several studies suggest that plasma treatment may reduce the biological activity of mycotoxins by modifying critical functional groups, information regarding the identity, stability, bioavailability, and long-term toxicity of degradation products remains very limited. Comprehensive toxicological evaluation, therefore, is one of the main priorities for future research.

From an industrial perspective, scaling CP technology from laboratory systems to commercial food-processing operations remains challenging. Uniform treatment of heterogeneous bulk materials, such as grains, nuts, and dried fruits, is difficult because of irregular surfaces, particle packing, and the limited penetration depth of reactive species. Future research should therefore prioritize pilot-scale validation, the optimization of reactor design, improved energy efficiency, and integration into existing processing lines. Atmospheric-pressure systems using air as a feeding gas may offer a more practical and economically achievable option for industrial applications.

Regulatory acceptance also remains limited due to insufficient information regarding process reproducibility, possible byproduct formation, toxicological safety, and long-term effects on food quality. The generation of harmonized datasets obtained under realistic industrial conditions will be essential to support future regulatory approval and commercial adoption.

Overall, the main take-home message of this review is that CP technology shows considerable potential as an alternative strategy for mitigating fungal contamination and mycotoxins in low-moisture foods, particularly because it can achieve effective decontamination while preserving product quality under optimized conditions. However, wider industrial implementation will require further progress in process standardization, mechanistic understanding, toxicological assessment, pilot-scale validation, and regulatory harmonization.

## Figures and Tables

**Table 1:** Comparison of the main CP sources, production mechanisms, key parameters, and their advantages/drawbacks for food applications.

CP Device	Mechanism	Key Parameters for Food Decontamination	Key Applications in Food Preservation	Advantages	Drawbacks
Dielectric Barrier Discharge (DBD)	High voltage between parallel electrodes separated by a dielectric material creates micro-discharges.	Type of gas; ambient air; N_2,_ Ar, He, or mixtures of noble gases with N_2_ or O_2_; Voltage: usually 5–30 kV peak-to-peak; Power: 1–500 W; Frequency: 50 Hz–10 kHz; Distance between electrodes: 1–10 mm gap; out of gap (indirect): up to a few cm. Treatment time: usually 1–10 min.	Surface decontamination of nuts, grains, seeds, and spices. Degradation of mycotoxins. DBD may be applied directly with the target in the gap between electrodes (in-package) or indirectly. The parameter values strongly influence the outcomes in foods.	Highly uniform, low energy consumption, scalability, and works with air at atmospheric pressure. Effective for large, flat food surfaces or in sealed packages. Flexible chemistry by changing the gas composition and thus the proportion of ROS/RNS.	Limited penetration depth to kill microorganisms on irregular food surfaces. It can cause lipid oxidation and also color changes over long exposure times. Potential for ozone accumulation; high initial cost for scalability and standardization.
Atmospheric Pressure Plasma Jet (APPJ)	A gas flows through high-voltage electrodes, creating a focused beam through a nozzle (plume or jet).	Type of gas: Ar, He, compressed air, O_2_, N_2_. Can work at atmospheric pressure; flow 0.1–5 L/min Voltage (AC or pulsed DC): 1–15 kV with noble gases; 15–30 kV with air or N_2_. Up to 90 kV for bulk grains in industry. Power: 0.5–50 W; Frequency: high, from 1–100 kHz to MHz (RF); Distance nozzle/target: 1–30 mm; Treatment time: 1–20 min (depending on the target).	It is used to treat dried foods like spices and nuts, and also fresh food, to eliminate bacteria and fungi. It can be applied to packaging materials, such as polymers (PET, PP).	Targeted application for specific spots reaching grooves, low gas temperature; versatile configurations. Ability to sustain a stable and homogeneous plasma discharge at atmospheric pressure. Low cost if used with air. Low thermal impact, like DBD. Easy adjustment of the distance to the target. Highly efficient for generating PAW.	Small treatment area (requires scanning); noble gases (Ar/He) have high cost, short lifetimes, and limited bactericidal and fungicidal effects. Air is difficult to control and needs high energy. More operational cost than air-based DBD. Main applicability in laboratories. Difficult to apply to bulk food in the industry.
Corona Discharge Plasma	The ionization process occurs near sharp electrodes where the electric field is strongest. High voltage accelerates free electrons in the air, which collide with neutral gas molecules of O_2_ or N_2_. Reactive species are generated near the high-field region and transported to the target.	Type of gas: usually ambient air or mixtures of O_2_/N_2_; Voltage: 1–10 kV (lab usage), 10–50 kV (for industry); Power: low, 0.1–100 W and low current; Frequency: low (50–500 Hz); Distance to target: 1–50 mm; treatment time: 0.5–30 min (depending on the target).	Decontamination of fruits, vegetables, and meat, and also low-moisture foods; sanitization of packaging materials; modification of surface properties; degradation of mycotoxins without thermal damage.	Simple, cost-effective, and easy setup (needle-to-plate/wire-to-plate); can be used in open-air; effective for surface sterilization. Lower power consumption than DBD. Efficient O_3_ generation.	Plasma distribution is often concentrated near electrodes, being non-uniform; best suited for localized, non-uniform, or smaller surface areas. This limits its applicability for large-scale, uniform, bulk food processing. Sensitive food products can face quality problems (fat oxidation) if treatment is not controlled. Less short-lived species reach the sample compared to DBD or APPJ.
Gliding Arc (GA) Plasma	The discharge forms between divergent electrodes, pushed by a gas flow, resulting in high concentrations of ROS.	Type of gas: usually air, O_2_, or N_2_; sometimes Ar; works at atmospheric pressure Voltage: usually 2–10 kV; Power: high, 100–5000 W; Frequency: usually 50–60 Hz (AC) or DC sources; Distance: 10–100 mm Treatment time: usually 1–6 min or up to 20–30 min (depending on the target).	Useful for pretreatment for hot-air drying of waxy fruits because it enhances dehydration efficiency by changing surface properties and creating micro-holes. Inactivates bacteria and fungi on the surface of fresh fruits, vegetables, and nuts. Extends the storage life of fresh produce; inactivates enzymes like peroxidase and catalase. Often used indirectly.	High efficiency for degrading mycotoxins like AFB_1_ and OTA; suitability for large-scale industrial applications; it uses low-cost gases, such as air, avoiding toxic chemicals.	It can produce high temperatures if it is not accurately controlled (power, time). Long exposure times can cause darkening, surface etching, and excessive oxidation, affecting the color and appearance of food products. Initial investment is high. Not appropriate for direct application to foods
Microwave (MW) Generated Cold Plasma	Generated by microwave sources. High-frequency electromagnetic fields ionize gases at or near room temperature. Systems can operate at low or at atmospheric pressures; usually, electrodes are not needed. Magnetrons can be substituted with other MW-generating systems.	Type of gas: ambient air, Ar, N_2_, O_2_, and mixtures of Ar + O_2_ or N_2_ + O_2_; Voltage: low but high power (200–6000 W); Frequency: 2.45 GHz; Distance: 5–50 mm; Treatment time: 1–30 min.	Surface decontamination of fresh fruits, vegetables, meat, and seafood. Shelf-life extension for chicken, pork, and fishery products. Inactivation of fungi and mycotoxins on nuts, cereals, and spices; In-package or package-surface disinfection.	High electron density with relatively low energy; good for uniform surface sterilization; can be operated without electrodes; rapid sterilization (often <5 min) of microbial contaminants, generally easier to scale for industrial applications.	Higher setup/capital costs; challenging to control for non-uniform, complex food surfaces.
Fluidized Bed Plasma	An electrical discharge (DBD, APPJ) is used to create plasma within a bed of particulate food suspended in a gas stream, creating a fluid-like state. The flow rate controls the fluidization quality. High flow rates warrant maximum mixing.	Type gas: air, Ar, He, or N_2,_ or mixtures of O_2_, N_2,_ and CO_2_ Voltage: 10–20 kV; Power: 200–600 W; Frequency: 10–50 kHz. Distance CP to target: usually 3–10 mm; Treatment time: 5–60 min, with high effectiveness at 15–20 min.	Uniform treatment allows gas and RONS to reach the entire surface of each grain, overcoming the limitations of static plasma. Decontamination of grains, nuts, spices, and powdered foods.	High efficiency compared to static plasma at killing foodborne pathogens, attaining large reductions on grainy surfaces. Eco-friendly and energy-efficient. It preserves quality with minimal damage to texture, color, and nutritional attributes. Safe and dry process.	High initial investment for equipment and potential high electrical power consumption. Potential for surface abrasion of fine particles. Potential to accelerate lipid oxidation in high-fat foods if not optimized. Difficulties in ensuring uniform, large-scale industrial treatment for varied particle sizes.
Radio Frequency (RF) Plasma	Plasma is produced by applying a rapidly oscillating electric field to a gas where free electrons react almost instantly to the alternating field, but heavy molecules do not. It can work at atmospheric pressure or, more usually, at low pressure.	Type of gas: ambient air, N_2_, mixtures of O_2_ + N_2_, or Ar + O_2_; Voltage: low pressure (100–1000 V), atmospheric pressure (0.5–10 kV); Power: 10–200 W; Frequency: low, 10–150 kHz up to 13.56 MHz. Distance plasma-target: variable depending on the reactor geometry, 5–50 mm; Treatment time: 1–15 or 20 min, depending on the type of food.	It directly breaks down chemical structures like AFs and OTA. It can denature surface enzymes that contribute to spoilage, helping to extend the total shelf-life of the treated grain or nut.	It creates a highly homogeneous and stable diffuse glow. Unlike the localized filaments in DBD or the moving arc in GAD or CD plasmas, it ensures every side of seeds/nuts receives an identical plasma dose. Attains deep penetration. Temperature stays very close to room temperature, preserving vitamins, colors, and flavors in nuts, seeds, or dried fruits. Achieves significant fungal reductions in seconds to minutes.	RF plasma typically requires a sealed vacuum chamber and vacuum pumps. It implies high operational costs. Poor depth penetration in foods. It is a batch process, difficult to scale up into industrial processing chains. Higher complexity than other plasma sources (DBD, glow discharge).
Glow-Discharge Plasma	This is produced by the application of a high voltage across two electrodes in a chamber filled with a gas (usually Ar) at low pressure (1–10 Torr). Atmospheric pressure can be used.	Type of gas: air, N_2_, O_2_, or Ar; Voltage: 500 V up to 20–30 kV; Power: 10–500 W; Frequency: low (50 Hz AC, 3–5 kHz) or high (40 kHz, 13.56 MHz RF); Distance to target: 2–15 mm; Treatment time: 0.5–30 min.	Highly effective at inactivating pathogens in fresh foods and nuts; extends the shelf life of perishable foods; reduces enzyme activity and browning and off-flavors in foods; effective in cleaning dried foods (spices, grains, seeds); used to sterilize food contact surfaces and packages without affecting their bulk properties.	It achieves higher log reductions of fungi and spores compared to other CP methods, inactivating a wide range of bacteria, viruses, and fungi with no hazardous residues; highly uniform treatment across the entire large surface of particulate products (nuts, grains); produces a high density of RONS.	It usually operates under low-pressure conditions and consumes higher energy per unit of treated product than other CP devices; it necessitates expensive, bulky vacuum chambers and constrains its use in continuous processing; ineffective for internal contamination; works at lower temperatures than GAD plasma. High-intensity, long-time exposure can lead to lipid oxidation, protein degradation, and textural alterations in lipid-rich foods. Incompatible with volatile/high-moisture foods.

**Table 2:** Effect of cold plasma on toxigenic fungi in nuts and dried fruits.

Commodity	Type of CP	CP Conditions	Assayed Fungi	Effects on the Fungi	Ref.
Pistachios	Atmospheric pressure capacitive coupled plasma (AP-CCP)	Gas: Ar; Power: 50, 75, 100, and 150 W; Frequency: 13.65 MHz; Time: 2, 6, and 0 min	*A. flavus*	4 log fungal reduction (about 67%) was obtained at a power of 100 W for 10 min. At 150 W for 10 min, the fungus was eliminated. However, problems with texture were noticed.	[81]
Pistachios	AP-CCP DC-DP ICP	Gas: Ar; power: 50, 75, 100, 150 W; time: 2, 6, and 10 min; Gas: Ar at pressure: 2 Torr.; power: 50–300 W; time: 5–20 min; Gas: Ar; power: 250 W; time: 20 min	*A. flavus*	AP-CCP: 6 log reduction at 150 W and 10 min. DC-DP: 5 log reduction at 300 W and 20 min. ICP: 2 log reduction at 250 W and 20 min.	[82]
Pistachios	DBD	Gas: Environmental air; Voltage: 15 kV; Power: 130 W; Frequency: 20 kHz; Distance: 3 mm; Time: 0.25–3 min	*A. flavus*	4 log CFU/g reduction. Total inactivation of *A. flavus* at 3 min.	[83]
Pistachios	Plasma jet	Gas: Ar/Air 62.23%; Voltage: 20 kV; Time: 15 min	*A. flavus*	5.04 log CFU/g reduction after a plasma exposure for 15 min.	[6]
Hazelnuts (inoculated)	Atmospheric pressure fluidized bed (APFBP) plasma. Two reactors	Gas: dry air; Voltage: 5–10 kV; Max. power: 655 W; Frequency: 18–25 kHz; Time: 5 min; two reactor diameters (49 and 65 mm)	*A. flavus* and *A. parasiticus*	*A. flavus* reduction: 4.5 log cfu/g. *A. parasiticus* reduction: 4.19 log cfu/g.	[70,80]
Hazelnuts	DCSBD	Gas: N_2_, O_2_, synthetic air (with and without water vapor), ambient air; Time: 180 s	*A. flavus* (initial population 4.6 CFU/g)	*A. flavus* reduction: 1 log CFU/g (O_2_ and synthetic air with vapor). 2.56 log (synt. air without vapor). 2.95 log (ambient air). 1.69 log (N_2_).	[84]
Hazelnuts	AP plasma LP plasma	Gas: N_2_, air at 3000 L/h; Power: 655 W; Frequency: 25 kHz, 5 cycles; Time: 1.7 min; Distance: 7 cm	*A. flavus* and *A. parasiticus*	*A. flavus* reduction: 5 log CFU/g (N_2_); 5.4 log CFU/g (air). *A. parasiticus* reduction: 5 log CFU/g (N_2_). 5.5 log CFU/g (air).	[85]
		Gas: N_2_, O_2_, air; Power: 100 W; Frequency: 13.56 MHz; Time: 30 min; Distance: 10 cm		*A. flavus* reduction: 4.6 log CFU/g (N_2_); 4.5 log CFU/g (O_2_), 4.7 log CFU/g (air). *A. parasiticus* reduction: 4.4 log CFU/g (N_2_), 4.7 log CFU/g (O_2_), 5.6 log CFU/g (air).	
Walnuts (fresh and dried)	Plasma jet	Gas: Ar; Voltage: 15 KV (pulsed DC); Frequency: 12 kHz; Distance: 15 mm; Time: 0, 3, 5, 7, 9, 10, and 11 min	*A. flavus*	*A. flavus* elimination at 11 min in fresh walnuts. *A. flavus* elimination at 10 min in dried walnuts. 11 min of the treatment did not affect the total phenolic content of the fresh and dried walnuts.	[86]
Peanuts	Low-pressure capacitively coupled RF glow discharge.	Gas: atmospheric air, RH: 45.3 ± 0.3%; 0.2 mbar pressure; Voltages and powers: 1500 V at 40 W; 1950 V at 60 W; Frequency: RF, 13.56 MHz; Time: 0–30 min	*A. parasiticus* and *A. flavus*	Inactivation % of *A. parasiticus* and *A. flavus* increased with both time and power and were 99.9% and 99.5%, respectively.	[4]
Peeled peanuts	Plasma jet	Gas: 9 L/min Ar + 90 mL/min O_2_; Frequency: 80–800 kHz; Power: 20 W; Distance: 20 mm; Time: 10 min	*A. flavus*	100% growth inhibition (initial inoculation: 50 µL of 5 × 10^9^ spores/mL).	[76]
Peanuts	Rotary plasma jet	Gas: Ar at atmospheric pressure; High voltage (not specified) Power: 180 or 200 W; Frequency: 12.5 MHz; Time: 2.5–10 min	*A. flavus* and *A. niger*	At 5 min, the reductions in *A. niger* were 5.30 or 5.76 log CFU/g, at 180 or 200 W, respectively. At 3.5 min, the reductions in *A. flavus* were 4.08 or 4.64 log CFU/g at 180 or 200 W, respectively.	[87]
Peanuts (inoculated)	HVCAP	Gas: 78% N_2_; 22% O_2_ at three relative humidity (RH) values (5, 40, and 80%); Voltage: 90 kV; Time: 2, 5, and 10 min	*A. flavus*	The treatment time, RH, and post-treatment storage affect the reduction in *A. flavus* spores. Increasing the exposure time, RH, and post-treatment time increased the mold reduction rate. DBD-HVCAP, with a direct plasma exposure time of 10 min, at 90 kV, 80% RH air, and 24 h post-treatment storage time, can reduce 99.9% (3.50 log CFU/sample) of *A. flavus* spores in the peanut kernels.	[88]
Roasted coffee beans	DBD	Gas: He/air at 1.5 L/min flow; Voltage: 850 V; Power: 30 W; Frequency: 13.56 MHz; Time: 0–18 min	*A. niger*, *A. westerdijikiae*, *A. steynii*, and *A. versicolor*	4 log CFU/0.1 g roasted coffee beans reduction after 6 min of exposure.	[89]
Dried palm dates (inoculated)	Double atmospheric pressure argon cold plasma (DAPACP) jet	Gas: Ar at flow rates of 1.5, 2.5, 3.5, and 4.5 L/min; Sinusoidal high-voltage (15 kV peak-to-peak); Distance: 12 mm; Times: 0.5, 1, 2, 3, 4.5, 6, 7.5, and 9 min	*A. niger*	The best flow rate was 3.5 L/min. Working at this flow rate, conidia reduction was from 10^3^ to 20 CFU/100 mm^2^ of sample at 7.5 min (about 1.7 log reduction). Total inhibition was found at 9.0 min.	[90]

**Table 3:** Effect of cold plasma on toxigenic fungi in cereal grains.

Cereal	Type of CP	CP Conditions	Assayed Fungi	Effects on the Fungi	Ref.
Barley grains	DCSBD 400	Gas: dry air, 100% CO_2_, 80% CO_2_ + 20% O_2_) gas flow rate: 10 L/min; Power: 350 W; Time: 1 or 3 min	*A. niger* and *P. verrucosum*	*A. niger* and *P. verrucosum* reduction in counts by 2.5–3 log cycles (dry air) after two weeks.	[93]
Barley grains	Gliding arc discharge (GAD) plasma jet	Gas: air at 5 L/min; Voltage: 3.5 kV; Power: 8.5 W; Frequency: 19.2 kHz; Distance nozzle-target: 0.5 cm; Time: 0–30 min	*F. graminearum*	The reduction of 1 log CFU/g after the 20 min exposure demonstrated that this technology could possibly aid in controlling strategies for *F. graminearum*.	[65]
Maize grains (inoculated)	APFBP with two reactors	Gas: dry and filtered air and N_2_ at atmospheric pressure; flow-rate: 3000 L/h; Voltage: 10 kV; Power: 460–655 W; Frequency: 18–25 kHz Time: 1–5 min; Reactor diameters: 49 and 65 mm	*A. flavus* and *A. parasiticus*	Significant reductions of 5.48 and 5.20 log CFU/g in *A. flavus* and *A. parasiticus*, respectively, were achieved on maize grains after 5 min treatment in the first fluidized bed plasma reactor (diameter: 49 mm). *A. flavus* was more sensitive than *A. parasiticus* to plasma treatment at all tested conditions.	[2]
Maize grains	DCSBD	Gas: air Voltage (sinusoidal): 20 kV amplitude Power: 400 W; Frequency: 14 kHz; Time: 30–300 s	*A. flavus*, *Alternaria alternata*, and *F. culmorum*	Reduction of 3.79 log CFU/g in *F. culmorum* after a 60-s treatment, 4.21 log CFU/g in *A. flavus*, and 3.22 log CFU/g in *A. alternata* after a 300-s treatment.	[94]
Maize grains	DBD	Gas: ambient air, 50–55% RH; Voltage: 120–160 kV; Frequency: 50–70 Hz; Distance: 30 mm; Time: 2, 4, 8, and 12 min	*A. flavus*	Reduction of *A. flavus* spores by 0.96–3.20 log CFU/g at 8 min. 50 Hz was the best frequency to reduce the spore population	[7]
Maize grains	DBD using treatment approaches: close plasma treatment (CP), plasma-treated gas (PTG)	Gas: pure O_2_ and synthetic air; Voltage: 13 kV; Frequency: 6 kHz; Distance: 2 mm; Time: 3 and 20 min	*A. flavus* and *F. proliferatum*	Air-fed plasmas were the most effective against *A. flavus* and *F. proliferatum*. At 20 min, the reduction of conidial germination/growth by air-fed plasmas was 50% for *A. flavus*, and for *F. proliferatum*, this reduction was 90%	[55]
Wheat grains and in vitro	Dielectric barrier surface micro-discharge (SMD)	Gas: air at 1 L/min; Voltage: 4 kV; Power: 5 ± 0.15 W; Frequency: 7 kHz; Distance: 3 mm; Time: 0–3.5 min (in vitro); 3, 6, 9 min (wheat grains)	*F. graminearum* (two strains), *F. pseudograminearum*, *F. moniliforme*	Fungal inhibition of all strains:100% at time ≥3 min (in vitro using water). Reduction of only 0.4 log in wheat (3 min).	[79]
Wheat grains	Gliding arc cold plasma	Gas: dry air (21% O_2_, 79% N_2_); 2, 2.5, 3, and 3.5 kV; Power: 5.39, 6.88, 8.98, and 10.88 W; Frequency: 20 kHz; Time: 2, 6, and 12 min	*A. flavus* and *A. parasiticus*	57% increase in lag time, 68% reduction in growth rate, and 78% reduction of *A. flavus*. 70% increase in lag time, 55% reduction in growth rate, and 68% reduction of *A. parasiticus*. Higher potency and time both increase effectiveness.	[47]
Rice grain	DBD	Gas: not specified; Voltage: 25 kV; Distance: 20 mm; Time: 2, 4, 6, and 8 min	*A. niger*, *Rhizopus oryzae*, *Penicillium verrucosum*, and *F. graminearum*	A decrease of 65–80% in spore formation was observed.	[10]
Rice grain (contaminated)	DBD	Gas: 70% N_2_ + 30% CO_2_/25% O_2_ + 45% N_2_ + 30% CO_2_/45% O_2_ + 25% N_2_ + 30% CO_2_, and 65% O_2_ + 5% N_2_ + 30% CO_2_; Voltage: 70–100 kV; Time: 0–60 min	*A. parasiticus*	A 99.98% degradation rate against *A. parasiticus* was achieved at 90 kV after 5 min. CP could reduce the initial concentration of 6.05 to 2.28 log CFU/mL in 4 min. After 6 min of treatment, the fungus was completely eradicated. Moisture content of rice, treatment time, voltage, and gas composition significantly affected the decontamination rate.	[95]
Milled rice, paddy rice, and rice bran	MINI ACP high-voltage, low-frequency transformer discharge.	Gas: air; Voltage: 10 kV (max.) operated at ~2.7 kV output; Power: 5 W; Frequency: 30 kHz; Time: 5, 10, 15, and 20 min	*A. fumigatus*, *F. semitectum*, *F. moniliforme*, *F. solani*, *F. oxysporum*, and *F. poae*	>99% inactivation of *A. fumigatus* in milled and paddy rice, with slightly lower efficacy in rice bran (60% reduction in 10 min). *F. moniliforme* and *F. poae* were completely inactivated at 20 min. The remaining species were less sensitive (1–1.9 log reduction at 20 min in milled rice)	[8]
Buckwheat grains	Low-pressure radiofrequency CP (ICP)	Gas: O_2_ at low pressure (approx. 30 Pa); Radiofrequency: 27.12 MHz; Power: about 1.8 kW; Time: 0.5, 1, 2, and 3 min	*Alternaria alternata*, *A. flavus*, *A. niger*, *Cladosporium cladosporioides*, *Epicoccum nigrum*, *Fusarium fujikuroi*, *F. graminearum*, *F. oxysporum*, *F. proliferatum*, and *F. sporotrichioides*	After 2 min, *A. alternata*, *A. flavus*, *C. cladosporioides*, *E. nigrum*, and *F. graminearum* were completely inactivated. A few grains remained contaminated with *A. niger*. At 3 min, the most resistant fungi were *F. fujikuroi* (4 log CFU/g) and *F. proliferatum* (2.93 log CFU/g), followed by *F. sporotrichioides* and *F. oxysporum*.	[96]
Brown rice cereal bar	CAP jet	Gas: Ar, flow rate of 10 L/min; Max. Voltage: 10 kV; Power: 0–40 W; Time: 5, 10, 15, and 20 min	*A. flavus*	A power of 40 W during 20 min was required to reduce *A. flavus* growth of approximately 4 log CFU/g. The highest power and exposure time provided the highest fungal reduction.	[97]

**Table 5:** Effect of cold plasma on mycotoxins in nuts and dried fruits and in vitro.

Commodity	Type of CP	CP Conditions	Mycotoxins	Effects of CP on Mycotoxins	Ref.
Pistachios powder and in vitro	DBD	Gas: ambient air; Voltage: 15 kV; Power: 130 W; Frequency: 20 kHz; Distance: 3 mm; Time: 0.25, 0.5, 1, 1.5, 2, 2.5, and 3 min.	AFB_1_ (383 ppb)	-In pistachios, the maximum AFB_1_ reduction (52.42%) was observed after 3 min. -In glass slides (in vitro), the maximum AFB_1_ reduction was 64.63% after 3 min. AFB_1_ reduction increased with time.	[83]
Pistachios	Plasma jet	Gas: Ar/air mixture, flow-rate: 3 L/min; Voltage: 10, 15, and 20 kV; Frequency: 10 kHz; Time: 5, 10, and 15 min.	AFs (10, 15, and 20 ppb)	Experimental AF reductions: AFB_1_, 80%; AFB_2_, 75.5%; AFG_1,_ 41.5%; AFG_2_, 50.75% at 20 kV, after 15 min time, 50% Ar in the gas mixture, with 20 ppb of each AF.	[6]
Whole and chopped spiked pistachios, certified slurry pistachios, and in vitro standards	SDBD	Gas: air at 2 regimes: NOx (100% power) and O_3_ (10% power); Voltage: 6 kV; Frequency: 23 kHz; Power: 425.35 W (NOx)/42.54 W (O_3_); Distance: 4 and 20 cm; Time: 0–60 min.	AFs and OTA (standards)	The O_3_ regime was more effective than the NOx regime. Reduction rates in vitro were: AFB_1_ and AFG_1_ (99%), AFB_2_ and AFG_2_ (60%), OTA (70%), at 60 min and 4 cm distance (best conditions). -AFB_1_ reduction: 5%, 20%, and 20% for whole, chopped, and certified slurry pistachios, respectively. -OTA reduction: 9%, 23%, and 31% for whole, chopped, and certified slurry pistachios, respectively.	[40]
Hazelnuts (dehulled) and in vitro	DBD	Gas: N_2_, and three N_2_/O_2_ mixtures (0.1% O_2_, 1% O_2_, and 21% O_2_); Power: 400, 700, 1000, and 1150 W; Time: 1, 2, 4, and 12 min.	AFs (AFB_1_, AFB_2_, AFG_1_ and AFG_2_) Initial concentrations in hazelnuts (ng/g): AFB_1_ (8.02); total AFs (36.1)	-In hazelnuts. Reduction in the concentration of total AF and AFB_1_ > 70% (at 12 min). Lowest residual levels: AFB_1_ (29.1% at 12 min and 1150 W); total AF (25.8% at 12 min and 1000 W). -In vitro. Total AF detoxification at 1000 W for a few minutes. Pure N_2_ was more effective than the mixtures with O_2_. AFB_1_ and AFG_1_ were more sensitive than AFB_2_ and AFG_2_, respectively.	[3]
Hazelnuts (spiked) and in vitro	AP-CP LP-CP	Gas: N_2_, air at 3000 L/h; Power: 655 W; Frequency: 25 kHz, 5 cycles; Time: 1.7 min; Distance: 7 cm. Gas: N_2_, O_2_, air, pressure < 0.25 mbar; Power: 100 W; Frequency: 13.56 MHz; Time: 30 min; Distance: 10 cm.	AFB_1_ + AFB_2_ in vitro: AFB_1_ level: 1–1000 ppb, Total AF level: 1.03–1030.9 ppb In spiked hazelnuts: AFB_1_ level 3 ppb, -Total AF (AFB_1_ + AFB_2_) level: 6 ppb	In vitro: -AP-CP: reduction in AFB_1_ level varied from 17% (1000 ppb) to 75% (50 ppb); reduction in total AF level varied from 22% (1030.9 ppb) to 92% (1.03 ppb). -LP-CP: reduction in AFB_1_ level varied from 37% (1000 ppb) to 90% (10 ppb); reduction in total AF level varied from 41% (103.1 ppb) to 81% (1.03–2.1 ppb). In hazelnuts: both plasma treatments reduced 72–73% of AFB_1_ and 69–70% of AFB_1_ in the presence of AFB_2_.	[129]
Peanuts	Bell jar type CP	Gas: atmospheric air, RH: 45.3%; Voltages and powers: 1500 V at 40 W; 1950 V at 60 W; Frequency: 13.56 MHz; Time: 0–30 min.	AFs produced in samples by: *-A. parasiticus* (initial levels of AFB_1_ and AFG_1_: 2.35 and 0.06 ng/g) *-A. flavus* (initial levels of AFB_1_ and AFG_1_: 9.84 and 0.95 ng/g)	*-A. parasiticus* samples: reduction in AFB_1_ level was >70% at 40 W, 15 min, and >90% at 60 W, 12 min. AFG_1_ level was <LOD at all conditions. *-A. flavus* samples: reduction in AFB_1_ level was >65% at 40 W, 15 min, and >95% at 60 W, 12 min. Reduction in AFG_1_ level was 50% at 40 W, 15 min, and AFG_1_ level < LOD at 60 W, 12 min.	[4]
Peanuts	Atmospheric pressure plasma jet (APPJ)	Gas. air at a flow rate of 107 L/min; Voltage: 4.4 kV; maximum power 650 W; Frequency: 70–90 kHz; two regimes: constant and agitated; Times: 0.5, 1, 1.5, and 2 min (constant)/3, 4, and 5 min (agitated).	AFs produced by inoculated *A. flavus*	After 5 min of agitated treatment, AF concentration was reduced from 64.14 to 39.6 ppb (38% reduction). After 2 min of constant treatment, AFs were reduced from 62.3 to 48.2 ppb (23% reduction).	[130]
Peanuts	Rotary plasma jet	Gas: Ar at atmospheric pressure; High voltage (not specified); Power: 180 or 200 W; Frequency: 12.5 MHz; Time: 2.5–5 min.	AFs (AFB_1_ and total AFs)	The highest inhibitory effect was observed in peanuts treated at 200 W for 5 min. No AF was detected before day 29 after treatment. Peanuts treated at 180 W for 3.5 min AF levels were >100 ppb on day 15 after treatment.	[87]
Peanuts	HV CAP	Gas. Air; Voltage: 90 kV; Time: 2, 5, and 10 min.	AFB_1_	71.3% AFB_1_ without post-treatment. The reduction in AFB_1_ was achieved with a treatment of 2 min and 80% RH. AFB_1_ also significantly increased with increasing treatment time, higher RH, and post-treatment storage (*p* < 0.05).	[88]
Peanuts (inoculated with *A. flavus*)	Open-air multipin-plane plasma reactor (a type of DBD)	Gas: air at atmospheric pressure; Voltage: 20, 25, and 30 kV; Frequency: 50 Hz; Times: 5, 10, and 15 min.	AFB_1_ (produced by inoculated *A. flavus*)	AFB_1_ levels decrease with increasing time and voltage. AFB_1_ reduction up to 82.1% at 30 kV and 15 min.	[130]
Roasted coffee beans	DBD	Gas: He/air at 1.5 L/min flow; Voltage: 850 V; Power: 30 W; Frequency: 13.56 MHz; Time: 0–30 min.	OTA	50% OTA detoxification was achieved after 30 min of exposure to DBD. Toxicity for untreated roasted coffee was shown to be toxic, while toxicity for CP-treated coffee was reduced to “slightly toxic”.	[89]
Mixed nuts (naturally contaminated and spiked) and in vitro standards	AP-CP	Gas: air, pressure 2 bar; Voltage: 600 V; Frequency, 25 kHz; Distance: 9 cm; Time: 0, 2, 4, 8, and 16 min.	AFs	AF reduction: - spiked samples (16 min): AFB_1_ (88.4%); AFB_2_ (85.9%); AFG_1_ (54.63%); AFG_2_ (47.9%); total AF (72.6%). - natural samples (16 min): AFB_1_ (24.6%); AFB_2_ (14.6%); Total AF (22.7%). - in vitro (2 min): AFB_1_ (80.9%); AFB_2_ (42.1%), AFG_1_ (88.7%); AFG_2_ (52.1%); total AF (76.3%).	[9]
Date palm inoculated with *A. niger.*	Double atmospheric pressure argon cold plasma (DAP ACP) jet	Gas: Ar at flow-rates of 1.5, 2.5, 3.5, and 4.5 L/min; Sinusoidal high-voltage (15 kV peak-to-peak); Distance: 12 mm; Times: 0.5, 1.0, 2.0, 3.0, 4.5, 6.0, 7.5, and 9.0 min.	OTA and FB_2_	FB_2_ was not detected in the discs inoculated with 6-min plasma-treated *A. niger*. OTA was not detected when the fungus was treated for 7.5 min.	[90]

## Data Availability

No new data were created or analyzed in this study.

## Abbreviations

The following abbreviations are used in this manuscript

AP-CCP	Atmospheric pressure capacitively coupled plasma
AP-CP	Atmospheric pressure cold plasma
APFBP	Atmospheric pressure fluidized bed plasma
CAP	Cold atmospheric plasma
CCP	Capacitively coupled plasma
CDPJ	Corona discharge plasma jet
CP	Cold plasma
DAPACP	Double atmospheric pressure argon cold plasma
DDBD	Double dielectric barrier discharge
DBD	Dielectric barrier discharge
DC-DP	Direct current diode plasma
DCSBD	Diffuse coplanar surface barrier discharge
FFA	Free fatty acid
GAD	Gliding arc discharge plasma
HVACP	High-voltage atmospheric cold plasma
ICP	Inductively cold plasma
LP-CP	Low-pressure cold plasma
LTP	Low-temperature plasma
MDA	Malondialdehyde
N-APPJ/APPJ	Non-equilibrium atmospheric-pressure plasma jet
LTNP	Low-temperature nitrogen plasma
NTP	Non-thermal plasma
PAW	Plasma-activated water
PTG	Plasma-treated gas
RF	Radiofrequency
RMD	Reactive molecular dynamics
RNS	Reactive nitrogen species
RONS	Reactive nitrogen and oxygen species
ROS	Reactive oxygen species
SDBD	Surface dielectric barrier discharge
SMD	Dielectric barrier surface micro-discharge
TCA	Tricarballylic acid
